# Sex Disparities in Ischemic Heart Disease in South Asia: The Role of Dietary Factors

**DOI:** 10.1016/j.jacasi.2025.07.012

**Published:** 2025-10-16

**Authors:** Tania Rahaman, Edina Cenko, Olivia Manfrini, Angela Maas, Maria Bergami, Chris P. Gale, Martha Gulati, Raffaele Bugiardini

**Affiliations:** a Department of Medical and Surgical Sciences, University of Bologna, Italy; b IRCCS Azienda Ospedaliero-Universitaria di Bologna Sant’Orsola Hospital, Bologna, Italy; c Department of Women's Cardiac Health, Radboud University Medical Center, Nijmegen, Netherlands; d Leeds Institute of Cardiovascular and Metabolic Medicine, University of Leeds, Leeds, UK; e Barbra Streisand Women's Heart Center, Smidt Heart Institute, Cedars-Sinai Medical Center, Los Angeles, California, USA

**Keywords:** deaths, ischemic heart disease, prevalence, risk factors, sex differences, South-Asia

## Abstract

**Background:**

South Asia bears the highest global burden of ischemic heart disease (IHD). Understanding variations in IHD outcomes by sex, income level, and country can inform targeted public health strategies.

**Objectives:**

This study aimed to analyze sex-specific trends in IHD prevalence and mortality across South Asia.

**Methods:**

We conducted a cross-sectional analysis using Global Burden of Disease (GBD) study data from 2005 and 2021. Age-standardized mortality rates (ASMRs) for IHD and age-standardized prevalence rates (ASPR) were estimated for 5 South Asian countries (Bangladesh, Bhutan, India, Nepal, and Pakistan). The ASMR-to-ASPR index was calculated to assess the risk of death among individuals with IHD. Sex-based comparisons were performed using Z-scores with a 95% confidence threshold (Z = 1.96).

**Results:**

In all countries, men exhibited higher ASMR values than women (average: 167 vs 102 in 2005 and 190 vs 112 in 2021). The ASMR-to-ASPR index was higher in women than men only in Pakistan, in 2005 (4.3% vs 2.9%, respectively) and 2021 (4.4% vs 3.1%, respectively), indicating greater mortality risk among women with IHD. Z-score analysis comparing Bhutan (lowest female ASMR) and Pakistan (highest) revealed differences in deaths attributable to high systolic blood pressure (Z score: 3.30), low vegetable intake (Z score: 2.02), and low fiber intake (Z score: 2.00). These differences were not observed in men.

**Conclusions:**

Mortality among people with IHD remains high across South Asia, with sex disparities in outcomes observed primarily in Pakistan. Leading risk factors for IHD mortality among women include high systolic blood pressure and low intake of vegetables and fiber.

Studies primarily from high-income countries suggest that women may have a higher risk of death from ischemic heart disease (IHD) than men, yet limited data are available for low- and middle-income countries (LMICs).^1^^,^^2^ Trends in sex differences in IHD mortality vary significantly by region and over time, often reflecting disparities in health care resources.^3^ While globalization has brought improvements to health care systems in LMICs through expanded access to advanced medical technologies, it has also introduced Western lifestyles that contribute to increased cardiovascular risk factors. This dual influence raises questions about whether the sex differences observed in high-income countries can be extrapolated to LMICs, where health care challenges and lifestyle factors may differ considerably.

The U.S. Congress recently passed the South Asian Heart Health Awareness and Research Act (H.R.3771), introduced in June 2021 to enhance understanding of cardiovascular disease risk among Asian American subpopulations, where traditional risk recognition models may fall short.^4^ An analysis of cause-specific mortality data in the United States from 2003 to 2017 has indicated that, among Asians in the United States, Asian Indians exhibit the highest age-standardized mortality rates (ASMRs) from IHD across racial and ethnic groups.^5^ Similar findings, though varied among South Asian countries, have been observed in the U.K. Biobank,^6^ which showed elevated IHD risk in individuals of Bangladeshi and Pakistani origin residing in the United Kingdom. Yet, comprehensive data on sex disparities in IHD risk factors and outcomes remain lacking.

To better understand the increased IHD risk among South Asians, as well as potential sex differences in outcomes, we focused on South Asians residing in their countries of origin (Bangladesh, Bhutan, India, Nepal, and Pakistan) through the Global Burden of Disease (GBD) framework.^7^ The current study seeks to determine whether sex differences in IHD risk factor burden and prevalence are more pronounced in economically disadvantaged areas, if sex disparities in mortality following cardiovascular events are higher for women than men, or if both trends coexist. This approach allows for a deeper understanding of how sex and socioeconomic factors intersect to shape IHD outcomes among South Asian populations, with the goal of informing policymakers in designing tailored prevention and intervention strategies.

## Methods

### Data sources

This study is compliant with the Guidelines for Accurate and Transparent Health Estimates Reporting (GATHER) statement.^8^ We used mortality and population data from individual South Asian countries, specifically Bangladesh, Bhutan, India, Nepal, and Pakistan, as provided by the GBD study.^7^ These data were supplemented with economic classifications from the 2023-2024 World Bank data, which categorize South Asian countries as LMICs with gross national incomes (GNIs) per capita of $4,515 or less.^9^ We used the World Bank’s 2023–2024 classification because it is the most recent and reflects the current global economic landscape. Using the latest classification ensures comparability with contemporary studies and provides a stable reference point, minimizing inconsistencies that could arise from using outdated classifications from individual study years. Among these countries, Bhutan and Bangladesh had the highest GNI per capita, whereas Nepal ranked the lowest (Supplemental Table 1). Because the data analyzed are publicly available, with no personally identifiable information, ethical approval was not required for this study.

### Terminology

As sex and gender often interact, we consistently used the term "sex" throughout this paper to reflect biological differences under investigation.

### Time frame and trends

The analysis period was determined based on data availability from the GBD Mortality Database. Because of a reporting lag of 12 to 18 months after each calendar year, the most recent comprehensive data for South Asian countries were from 2021. We selected 2005 as the starting point to provide a sufficiently long time frame (16 years) to assess meaningful trends in IHD-specific mortality rates. This allows for the capture of long-term changes rather than short-term fluctuations. We compared IHD specific mortality rates between 2005 and 2021; we also examined yearly trends across all included countries, which represent diverse geographies and economic statuses.

### Risk factor estimation

We selected conventional risk factors for IHD based on evidence of causality, including high systolic blood pressure, elevated low-density lipoprotein (LDL) cholesterol, high fasting plasma glucose, tobacco use, and high body mass index (BMI). According to the GBD 2021 Risk Factors Collaborators,^10^ smoking exposure included current or previous use of any tobacco product, as well as second-hand smoke, with the theoretical minimum risk exposure level (TMREL) set at zero. TMRELs for other risk factors were set as follows: blood pressure 110 to 115 mm Hg, fasting plasma glucose 4.8 to 5.4 mmol/L, LDL cholesterol 0.7 to 1.3 mmol/L, and BMI 20 to 25 kg/m^2^.

In addition, nonconventional risk factors, such as low physical activity (<3,000-4,500 metabolic equivalent minutes per week) and exposure to ambient air pollution (ambient ozone, PM2.5, and household air pollution from solid fuel use) were assessed using similar standardization methods.

### Health effects of dietary risks

An unhealthy diet is a major, preventable risk factor for noncommunicable diseases in both women and men. We identified 13 dietary risk factors for IHD based on GBD selection criteria.^11^ These included inadequate intake of whole grains, fruits, fiber, legumes, vegetables, nuts and seeds, seafood omega-3 fatty acids, and polyunsaturated fatty acids (PUFAs), as well as excessive consumption of trans fats, processed meat, sugar-sweetened beverages, and sodium. Optimal intake levels for each factor are provided in Supplemental Table 2.

### Statistical analysis

We analyzed sex-specific outcomes for IHD, focusing on ASMRs, age-standardized prevalence rates (ASPRs), and the ASMR-to-ASPR ratio per 100,000 inhabitants from 2005 to 2021 across the South Asian countries. The ASMR-to-ASPR ratio evaluates whether a region or demographic group has higher or lower mortality relative to the disease’s prevalence.

The ASMR-to-ASPR index is, by definition, a unitless ratio derived from dividing 2 rates (ASMR and ASPR), both expressed per 100,000 population. For ease of interpretation, we multiplied the ratio by 100 and expressed it as a percentage, representing the proportion of individuals with IHD who die from the disease annually.

To evaluate sex disparities in IHD risk, we calculated women-to-men rate ratios for both population attributable fractions (PAFs) and the ASMR-to-ASPR index, where a ratio of 1.0 indicates equal risk, >1.0 indicates relatively higher risk for women, and <1.0 indicates relatively lower risk for women. Uncertainty intervals (UIs) for the ASMR-to-ASPR index and women-to-men PAF ratios were derived using GBD 2021 methodology. The lower UI bound reflects higher prevalence/lower mortality, whereas the upper UI bound represents lower prevalence/higher mortality. We standardized both ASMR and ASPR to the GBD standard population, as described in previous publications.^12^

To evaluate the burden of modifiable risk factors, we analyzed GBD-derived ASMRs attributable to specific exposures. These estimates are based on TMRELs and were modeled using the GBD and Burden of Proof meta-regression frameworks.^13^ Full methodological details and code sources are publicly available.^14^

Sex differences in IHD outcomes were assessed using 2-sample Z-tests for independent proportions. This approach was applied both to ASMR-to-ASPR indexes and to risk factor–attributable ASMRs. Bhutan, the country with the lowest ASMR in both sexes, served as the reference for Z-score analyses, with 95% (Z > 1.96) confidence thresholds. Descriptive comparisons were also made using women-to-men rate ratios, where values >1.0 indicate relatively greater female burden.

To assess trends and associations, we calculated Pearson’s correlation coefficient (r), with a *P* < 0.05 indicating statistical significance. The normality assumption for the Pearson correlation was tested using the Shapiro-Wilk test to ensure validity. All analyses were based on publicly available data from GBD and World Bank data. All analyses were performed using Stata version 17.0. Further details on statistical methods and adjustments are provided in the Supplemental Material.

### Role of the funding source

This research did not receive additional support from organizations beyond the authors’ academic institutions. Therefore, no funders had any role in the study design, data collection, data analyses, data interpretation, or writing of the report.

## Results

### Mortality

**Figure 1:**
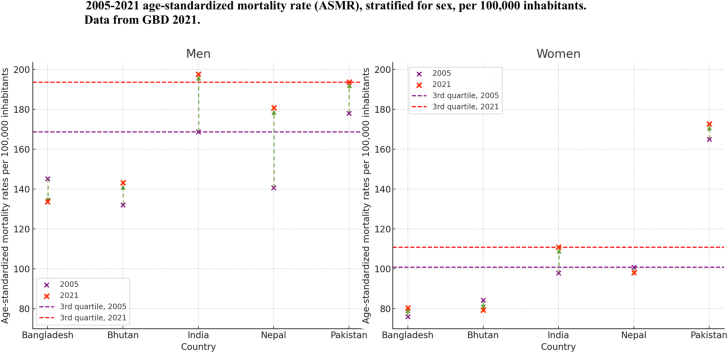
Trends in ASMR for IHD (2005 vs 2021) Trends in age-standardized mortality rates (ASMRs) per 100,000 inhabitants for ischemic heart disease (IHD), stratified by country and sex, comparing 2005 and 2021 using GBD (Global Burden of Disease) 2021 data. Mortality rates are represented by crosses, with purple indicating 2005 and red representing 2021. Horizontal lines denote the third quartile of ASMR for 2005 (purple) and 2021 (red). Arrows indicate the direction of change: upward arrows for increasing mortality trends and downward arrows for declining mortality trends.

In South Asia, between 2005 and 2021, ASMR increased significantly for both men and women (absolute rate difference 13.7% and 10.3%, respectively), as shown in Figure 1 and Supplemental Table 3. By 2021, men continued to carry the majority of the ASMR burden, with ASMRs reaching 848 deaths per 100,000 for men compared with 541 deaths per 100,000 for women. Specifically, high ASMRs for men (equal or exceeding the 3rd quartile for 2005; 169 deaths per 100,000) were seen in India and Pakistan. Similarly, women in these countries recorded the highest rates among the South Asia group of countries, with figures exceeding 101 deaths per 100,000. Throughout the study period, India and Pakistan reported the highest (above the 3rd quartile for 2021) ASMRs, with more than 193 deaths per 100,000 for men and more than 111 deaths per 100,000 for women.

### Prevalence

**Figure 2:**
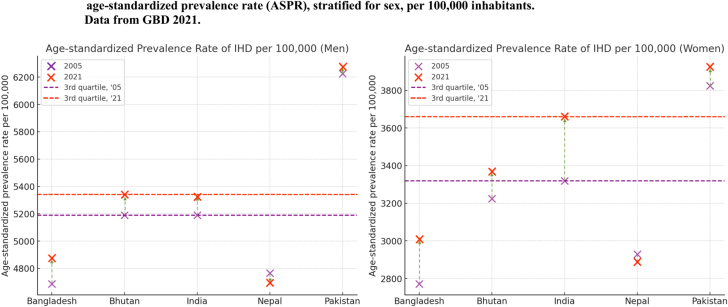
Trends in ASPR for IHD (2005 vs 2021) Trends in age-standardized prevalence rates (ASPRs) of IHD, stratified by country and sex, comparing 2005 and 2021 based on GBD 2021 data. Prevalence rates are represented by crosses, with purple indicating 2005 and red representing 2021. Horizontal lines denote the third quartile of prevalence for 2005 (purple) and 2021 (red). Arrows indicate the direction of change: upward arrows for increasing prevalence and downward arrows for decreasing prevalence. Other abbreviation as in Figure 1.

By 2021, the prevalence of IHD in South Asia had increased since 2005, reaching 5347.44 per 100,000 inhabitants in men, a 2.1% increase, and 3,609.07 per 100,000 in women, reflecting an 8.9% increase (Figure 2, Supplemental Table 3). Over the study period, Pakistan consistently reported the highest ASPR values, remaining above the 3rd quartile each year, with rates exceeding 5,189 per 100,000 in 2005 and 5,340 in 2021 for men, and 3,319 in 2005 and 3,659 in 2021 for women. Bhutan consistently ranked above the 3rd quartile for men in both 2005 and 2021, whereas India exceeded the 3rd quartile for women in both years.

### ASMR-to-ASPR index

**Figure 3:**
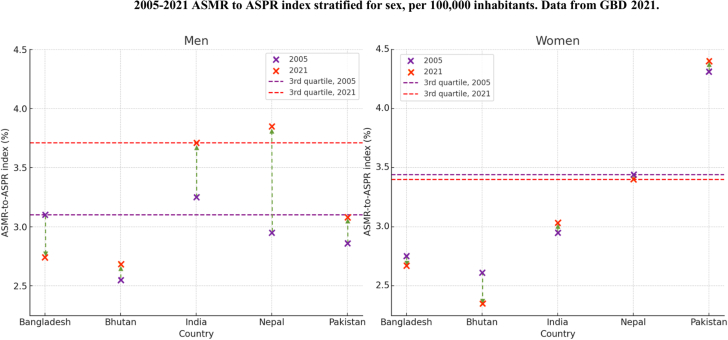
Trends in IHD Mortality Normalized by Prevalence (2005 vs 2021) Trends in IHD mortality normalized by its prevalence (ASMR to ASPR index), stratified by country and sex, from 2005 to 2021 using GBD 2021 data. The index is represented by crosses, with purple denoting 2005 and red representing 2021. Horizontal lines indicate the third quartile of the index for 2005 (purple) and 2021 (red). Arrows depict the direction of change: upward arrows for an increasing ASMR-to-ASPR index and downward arrows for a decreasing index. Abbreviations as in Figures 1 and 2.

Descriptive statistics stratified by sex for the ASMR-to-ASPR index in South Asian countries from 2005 to 2021 are presented in Figure 3 and Supplemental Table 3. During this period, the South Asian mean ASMR-to-ASPR index increased across all countries for both women and men. In 2005, the regional mean was 3.19% for men and 3.07% for women, increasing to 3.55% for men and 3.11% for women by 2021. However, these averages obscure notable country-specific differences. India and Pakistan showed a consistent increase in the ASMR-to-ASPR index for both sexes, whereas Bhutan exhibited an increase only in men, and Bangladesh saw a decrease in the index for both women and men.

### ASMR-to-ASPR index and GNI scores

Pearson correlation analysis showed a significant negative correlation between the ASMR-to-ASPR index and GNI per capita for both women and men (r = −0.77; *P* = 0.01), as shown in Supplemental Figure 1. This finding suggests that higher GNI per capita is associated with a lower ASMR-to-ASPR index, indicating a potential link between economic status and reduced IHD mortality relative to its prevalence.

### Women-to-men ratio in ASMR-to-ASPR index

**Figure 4:**
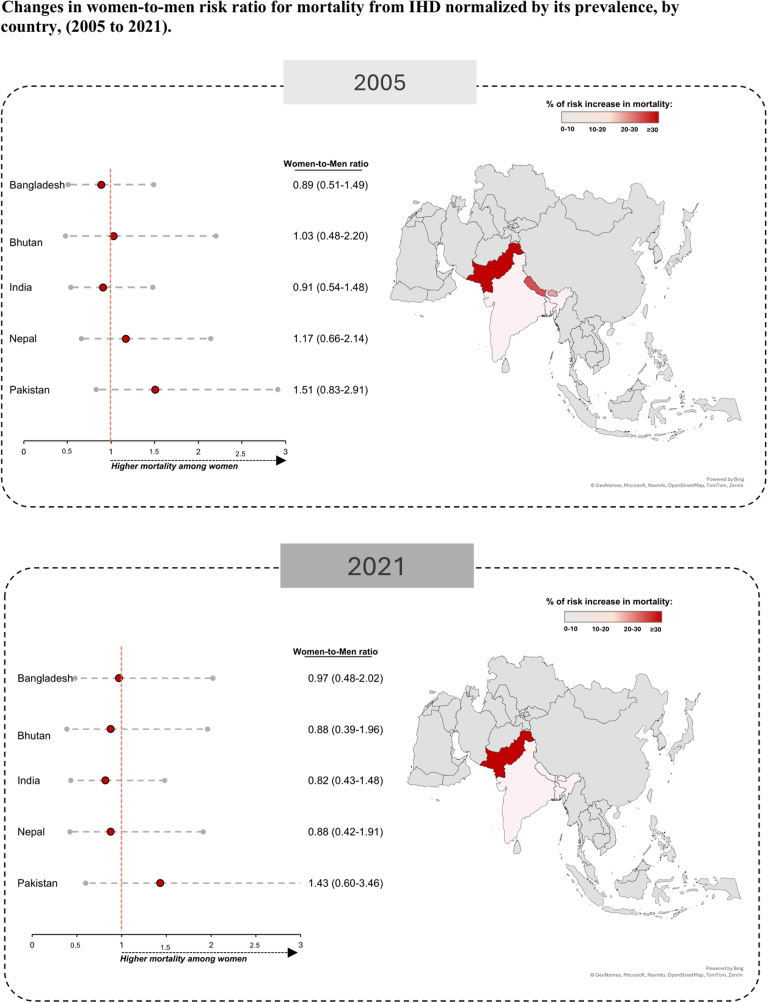
Women-to-Men Risk Ratio Change, Prevalence-Normalized (2005 vs 2021) Changes in the women-to-men risk ratio for IHD mortality, adjusted for its prevalence (ASMR-to-ASPR index), across South Asian countries from 2005 to 2021, using GBD 2021 data. The left panel displays risk ratios, with a red vertical line at 1.0, indicating equal mortality risk between sexes. Data points positioned to the right suggest higher mortality risk in women, whereas those to the left indicate higher mortality risk in men. The right panel presents maps showing the percentage increase in mortality risk, with darker shades representing greater increases over time. Abbreviations as in Figures 1 and 2.

In 2005, most South Asian countries exhibited a higher ASMR-to-ASPR index for women compared with men; however, this pattern was no longer prevalent by 2021 (Figure 4). In 2021, Pakistan was the only country with a women-to-men ASMR-to-ASPR ratio exceeding 1, reflecting a 43% higher mortality burden in women relative to men (Supplemental Table 3).

### Women-to-men ratio in ASMR-to-ASPR index and GNI scores

Pearson correlation analyses indicated no correlation between the women-to-men ratios in ASMR-to-ASPR index and GNI per capita in either 2005 or 2021 (r = –0.33, *P* = 0.36), as shown in Supplemental Figure 2.

### Z-score analysis of ASMR-to-ASPR ratios with Bhutan as a reference

The Z-score analysis for men in both 2005 and 2021 (Supplemental Table 4) found that none of the countries, Bangladesh, India, Nepal, or Pakistan, exceeded the significance threshold of 1.96 in ASMR-to-ASPR ratios compared to Bhutan, indicating no statistically significant differences at the 95% confidence level. In contrast, for women, Pakistan alone approached statistical significance, with Z-scores close to or exceeding 1.96, suggesting a potentially meaningful difference in the ASMR-to-ASPR ratio for women in Pakistan relative to Bhutan.

### Proportion of deaths attributable to conventional IHD risk factors

**Figure 5:**
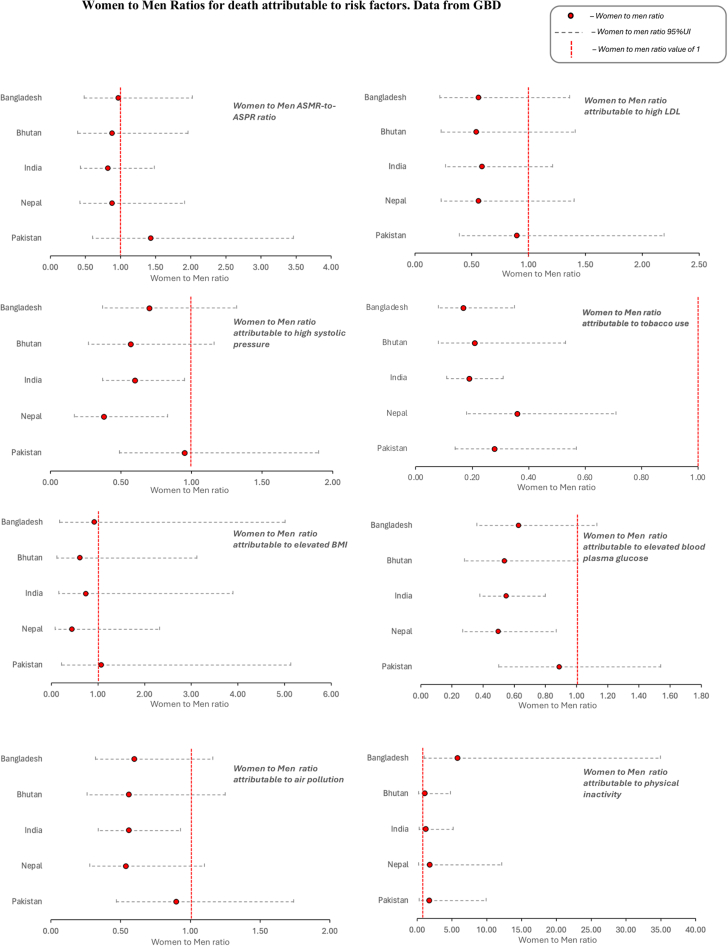
Women-to-Men Risk Ratios Attributable to Behavioral and Metabolic Risk Factors Women-to-men risk ratios for IHD mortality linked to behavioral and metabolic risk factors in South Asia, using GBD 2021 data. Red circles represent risk ratios, with grey horizontal line representing 95% uncertainty intervals. The red vertical line at 1.0 indicates equal mortality between sexes. BMI = body mass index; LDL = low-density lipoprotein; other abbreviations as in Figures 1 and 2.

Figure 5 presents the women-to-men ratios of population attributable fractions for deaths linked to the 5 conventional risk factors: high systolic blood pressure, elevated fasting plasma glucose, high LDL cholesterol, tobacco use, and elevated BMI in 2021. Across all South Asian countries, conventional risk factors generally resulted in higher mortality rates for men than for women, with the exception of elevated BMI in Pakistan (risk ratio [RR]: 1.07) (Supplemental Tables 5 to 10). Tobacco use exhibited the most pronounced sex gap, with an overall average women-to-men risk ratio of 0.24, showing minimal variation across countries. In contrast, metabolic risk factors displayed substantial variation in the degree of risk reduction observed in women across different factors and countries. For high LDL cholesterol, Pakistan showed the smallest sex difference, with a women-to-men risk ratio close to parity (RR: 0.90), indicating a relatively similar impact on IHD mortality between the sexes. For high systolic blood pressure, both Pakistan (RR: 0.95) and Bangladesh (RR: 0.70) recorded ratios above the average (RR: 0.64), indicating a smaller reduction in risk for women compared with men. Similarly, for elevated fasting plasma glucose, Pakistan (RR: 0.89) and Bangladesh (RR: 0.63) showed relatively higher women-to-men ratios, reflecting a closer mortality impact between women and men. In summary, Pakistan stands out among South Asian countries as having more comparable impacts of conventional risk factors on age-standardized IHD mortality rates between women and men, with risk ratios approaching parity across several factors.

### Proportion of deaths attributable to nonconventional IHD risk factors

Among nonconventional risk factors, physical inactivity showed the most pronounced sex differences in IHD mortality, disproportionately affecting women compared with men (Figure 5, Supplemental Table 11). Women-to-men risk ratios ranged from 5.81 in Bangladesh to 1.07 in Bhutan, indicating significant variability across countries. Particularly pronounced differences, with more than a 70% higher attributable risk for IHD mortality in women, were observed in Pakistan (RR: 1.74) and Nepal (RR: 1.79). In contrast, PM2.5 exposure posed a stronger risk of IHD mortality for men than for women across most South Asian countries. The narrower gap in Pakistan (RR: 0.90) aligns with the pattern observed for conventional risk factors indicating a relatively closer impact on mortality rates between men and women.

### Bhutan as a baseline for statistical significance using Z-Score analysis

Our Z-Score analysis highlighted significant sex-specific differences in IHD mortality across South Asian countries (Supplemental Tables 12 to 18). In Pakistan, although risk factors such as high systolic pressure, tobacco use, elevated fasting plasma glucose, and air pollution significantly contribute to IHD mortality, they affect women and men similarly and do not fully explain the observed sex disparity. High systolic pressure emerged as the primary driver of higher IHD mortality in Pakistani women, with a significant Z-score for women (3.30) but not for men (1.56). Among all countries analyzed, Pakistan was the only country where high systolic pressure had a significantly greater impact on women than men, underscoring its key role in the observed sex disparity in IHD mortality, as reflected by the ASMR-to-ASPR index.

### Dietary risk factors and their impact on mortality

**Figure 6:**
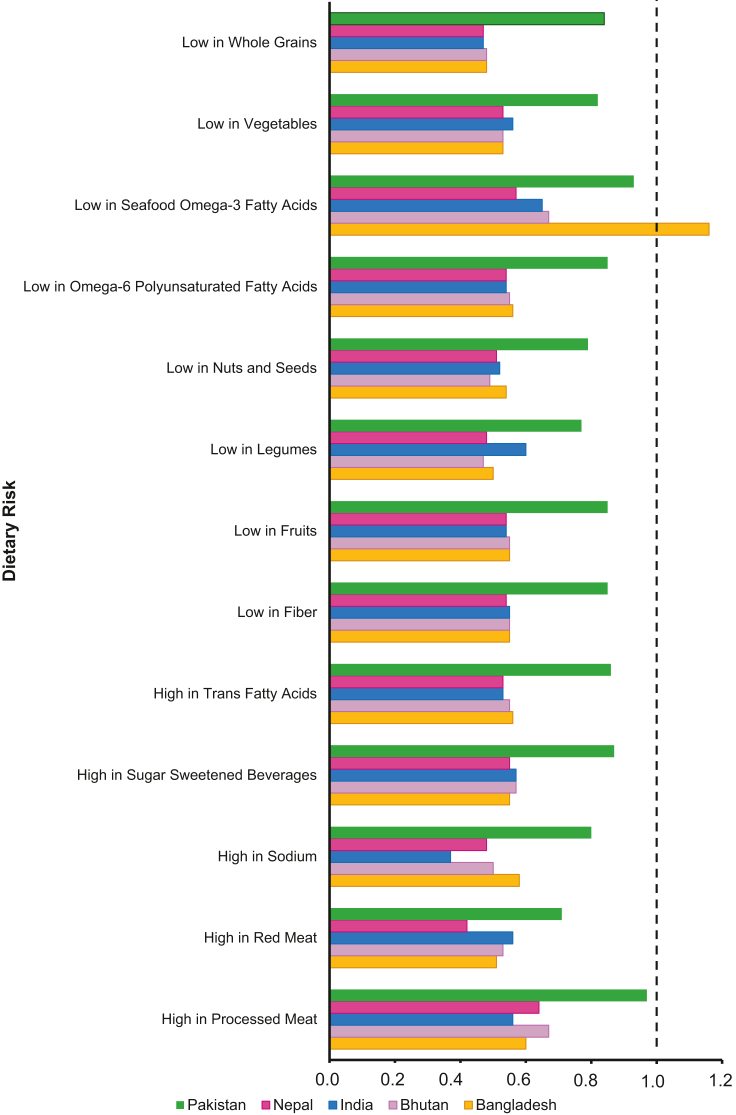
Women-to-Men Risk Ratios Attributable to Dietary Risk Factors Women-to-men risk ratios for mortality linked to dietary risk factors in South Asia, based on GBD 2021 data. The ratios highlight sex-specific differences in mortality attributable to dietary factors, including low vegetable and fiber intake. Risk factors are grouped with countries color-coded for clarity. Abbreviation as in Figure 1.

Globally, consumption of nearly all healthy foods and nutrients is suboptimal in South Asia, where men generally have a less healthy diet than women (Figure 6, Supplemental Tables 19 to 30). However, in Pakistan, the dietary gap between men and women is relatively small compared with other South Asian countries. Women consume 18% or less of the healthy foods and nutrients compared with men, including whole grains (16%), vegetables (18%), seafood omega-3 fatty acids (7%), omega-6 PUFAs (15%), fruits (15%), and fiber (15%). Similarly, the intake of unhealthy foods shows minimal differences between men and women, with gaps below 15% for trans fatty acids (14%), sugar-sweetened beverages (13%), and processed meat (3%).

### Diet-driven mortality: a comparative analysis using Z-scores

Our Z-score analysis comparing Bhutan and Pakistan revealed significant differences in the proportion of IHD deaths attributable to specific dietary factors. These differences were observed for whole grains in both women (Z-score: 3.47) and men (Z-score: 2.46), as well as for vegetables (Z-score: 2.02) and fiber (Z-score: 2.00) in women. These findings suggest that low intake of vegetables and fiber may play a key role in the adverse IHD mortality outcomes observed in Pakistani women.

### Generalizable population and variability in trends

The generalizable population for the GBD study is based on estimates provided by the World Bank, which uses the total population and age/sex distributions from the United Nations Population Division's World Population Prospects: 2022 Revision (Supplemental Table 31). To account for potential year-to-year variability that could make direct comparisons between 2005 and 2021 misleading, we conducted a detailed trend analysis of yearly ASMR, ASPR, the ASMR-to-ASPR index, and the women-to-men ASMR-to-ASPR ratio from 2005 to 2021 (Supplemental Tables 32 to 40). Only one country, Pakistan, exhibited a women-to-men ASMR-to-ASPR ratio consistently equal to or above 1.4 across all years, indicating a relatively higher risk for women or a relatively lower risk for men in that population.

We also examined the association between economic development and IHD burden by analyzing the Pearson correlation between GNI per capita and the ASMR-to-ASPR index during the period 2005–2021 (Supplemental Tables 38 and 39). The results revealed marked differences by country and sex. In men, India, Pakistan, Bhutan, and Nepal showed strong positive correlations (r > 0.7), whereas Bangladesh displayed a moderate inverse relationship (r = –0.54). Among women, correlations were more variable: Bhutan and Bangladesh showed strong inverse associations (r = −0.82 and −0.49, respectively), whereas Pakistan and India showed positive correlations (r = 0.72 and 0.35, respectively). These findings suggest that national income growth in the period 2005 to 2021 did not uniformly translate to improvements in IHD outcomes and may contribute differently to sex-specific disparities across countries.

## Discussion

Mortality among people with IHD has substantially increased across South Asian countries from 2005 to 2021. However, ASMRs per 100,000 inhabitants varied widely, ranging for men from 197 in India to 76 in Bangladesh, and for women from 172 in Pakistan to 79 in Bhutan. These patterns imply sex-specific differences in the worsening landscape of cardiovascular disease prevention and treatment across the countries of the region. To better evaluate sex disparities in IHD outcomes, we used the ASMR-to-ASPR index, which normalizes mortality to prevalence rates. This index gains insights into the likelihood of death among populations already affected by IHD, serving as a proxy for the effectiveness of measures aimed at preventing severe IHD manifestations as well as early diagnosis and treatment across sexes and countries. It also reflects the health care system's capacity to manage IHD in each population.

Two key findings emerged in our study (Central Illustration). First, the consistently higher mortality relative to prevalence (ASMR-to-ASPR index) in Pakistani women compared with men (4.31% vs 2.86% in 2005 and 4.40% vs 3.08% in 2021), unlike in other South Asian countries, suggests that factors beyond income level, which is relatively similar across these nations, are driving this disparity. Second, an analysis of upstream risk factors highlights metabolic and dietary contributors as major drivers of excess IHD mortality in Pakistani women. High systolic blood pressure and low intake of vegetables and fiber were identified as the most critical determinants of the elevated mortality risk in women compared with other countries in the region. These findings underscore the urgent need for targeted interventions to address these modifiable risks and reduce sex disparities in IHD outcomes in South Asia.

**Central Illustration:**
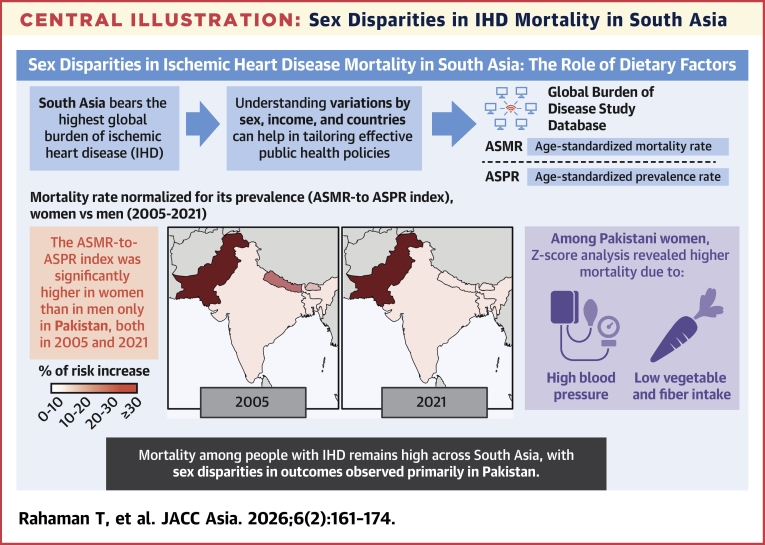
Sex Disparities in IHD Mortality in South Asia Regional sex disparities in ischemic heart disease (IHD) mortality across South Asia from 2005 to 2021, using Global Burden of Disease data. Shown are age-standardized mortality-to-prevalence ratios (ASMR-to-ASPR index), with maps showing that women in Pakistan consistently experienced higher mortality risk compared with men. Z-score analysis identified high systolic blood pressure and low vegetable and fiber intake as major contributors to excess mortality in Pakistani women. These findings underscore the importance of targeted dietary and metabolic interventions. Arrows indicate relationships between concepts. Darker map shading reflects higher relative mortality risk. Purple highlights key risk factors identified in Z-score analysis.

The observed decline in the average ASMR-to-ASPR index for South Asian women compared with men between 2011 and 2021 likely reflects the combined effects of targeted public health initiatives and advances in clinical care. Increased awareness of sex-specific differences in cardiovascular disease may have led to earlier diagnosis and improved management of IHD in women.^15^ Additionally, the expanded availability of statins, antihypertensives, and diabetes management therapies may have disproportionately benefited women, particularly given their historically lower treatment rates.^16^ Lifestyle-related changes, including greater awareness of physical activity and nutrition among women, may also have played a supporting role. However, although the narrowing of the ASMR-to-ASPR index gap is encouraging, the persistence of elevated female IHD mortality in specific regions, such as Pakistan, where mortality remains disproportionately high relative to prevalence compared with other South Asian countries, underscores the need for continued focus on structural barriers to equitable care.

The rising IHD burden in Pakistani women reflects the pervasive metabolic epidemic when compared with other South Asian countries. Across the region, high LDL cholesterol levels, elevated fasting plasma glucose, and high systolic blood pressure were more strongly associated with IHD mortality in men than in women. Similarly, air pollution and unhealthy diets had a greater overall impact on men. However, Pakistan stood out with the highest women-to-men ratios for these risk factors (RRs: 0.82 to 0.95) indicating a closer mortality impact between sexes compared with other South Asian countries. To further explore these disparities, we conducted a Z-score analysis comparing Bhutan (lowest ASMR for women) and Pakistan (highest ASMR for women). Significant differences were observed in IHD mortality attributable to high systolic blood pressure (Z-score: 3.30), low vegetable intake (Z-score: 2.02), and low fiber intake (Z-score: 2.00); differences were not observed in men. Taken together, these findings underscore the urgent need for targeted dietary and metabolic interventions to address modifiable risks and reduce the IHD mortality gap in Pakistani women.

Studies in the United States suggest ethnic group differences in hypertension prevalence, with Asians being at higher risk compared to their White counterparts.^17^ Similarly, among South Asian migrants to the United Kingdom, systolic blood pressure levels differ substantially compared with native populations.^6^ Among these groups, the population of Pakistan emerges as having the highest risk of hypertension. Prior studies have indicated several contributing factors, including low income, limited resources, lower education levels, systemic discrimination, and cultural influences in South Asian migrant populations.^18^ Yet, even after accounting for these disparities, much of the risk remains unexplained.^19^

Our findings reveal a striking difference in the prevalence of high systolic blood pressure between female populations in Bhutan and Pakistan, suggesting that this disparity cannot be explained solely by income or urbanization. Diet emerged as a key factor contributing to sex differences in hypertension and IHD-specific mortality. The introduction of Western dietary patterns through globalization or migration, marked by increased fat and energy intake, reduced carbohydrates, and a shift from whole grains to refined sources, has significantly lowered fiber and vegetable consumption, both linked to higher hypertension risk.^20^

Compounding this issue, cooking practices common in Pakistani households, such as deep frying, prolonged boiling, and the routine use of saturated fats such as ghee, can degrade the nutritional quality of vegetables and fiber-rich foods. These methods may reduce antioxidant and micronutrient content and can generate harmful compounds, such as advanced glycation end products, which have been linked to vascular inflammation and endothelial dysfunction.^21^ As a result, the expected cardiovascular benefits of diets nominally rich in vegetables may be attenuated or even reversed.

The role of diet may also shed light on the greater risk observed in women. Traditional unhealthy dietary habits, often resistant to change and deeply tied to social and cultural identity, are typically upheld by women as mothers and homemakers. This dynamic may inadvertently reinforce their adherence to traditional cuisine, increasing their susceptibility to hypertension and IHD mortality.

The interplay between low fiber and vegetable intake and high systolic blood pressure is well-documented.^22^^,^^23^ The beneficial effects of vegetables are thought to arise from their complex nutrient profile, which includes dietary fiber. Proposed mechanisms through which dietary fiber may influence blood pressure include its fermentation by gut microbiota, resulting in the production of short-chain fatty acids. These acids may exert vasodilatory effects by modulating the renin-angiotensin-aldosterone system.^24^ Furthermore, dietary fiber’s role in shaping gut microbiota composition could be critical for blood pressure regulation, as emerging evidence increasingly links gut dysbiosis to hypertension.^25^

Few large studies, primarily from high-income countries, have explored the association between diet quality and cardiovascular disease risk in women compared with men. The CGPS (Copenhagen General Population Study) has reported that poor diet was linked to an increased risk of cardiovascular disease in both sexes.^26^ In contrast, the PURE (Prospective Urban Rural Epidemiology) study found that an unhealthy diet was more strongly associated with cardiovascular disease in women than in men.^27^^,^^28^ These contrasting findings underscore the complexity of defining diet-related risk factors and their differential impacts by sex.

A limitation of these and other studies is their reliance on diet scores, which may obscure the contributions of individual dietary components to cardiovascular risk. Furthermore, differences in the selected components for constructing these scores add variability to the findings. The CGPS diet score included key items such as consumption of saturated and unsaturated fats, fruits, vegetables, fish, sugar-sweetened beverages, cold cuts like sausages and pâtés, and fast food. In comparison, the PURE study used a diet score based on 8 food types: fruits, vegetables, legumes, nuts, fish, dairy, unprocessed red meat, and poultry.

Although the GBD study has limitations, namely, its reliance on estimates derived from diverse data sources, it provides the most comprehensive analysis currently available. Importantly, GBD identifies clear relationships between specific dietary risk components and IHD occurrence, offering robust insights from a large dataset that encompasses LMICs, including South Asian countries.

### Study limitations

This study did not have individual-level data in reporting practices from many countries, along with variations in diagnostic and certification practices, which can influence the accuracy of cause-of-death determinations. Additionally, errors or biases in data collection, such as those from self-reported information, may affect the validity of the results. The use of survey-based data to estimate risk factor prevalence also presents challenges, as the accuracy of these estimates depends on the representativeness of the sample and the quality of the survey methods used.

Our analysis does not account for subnational variations, which can be substantial. As documented in the literature, ethnic subgroups within South Asia show significant differences in blood pressure levels, with systolic pressure being higher in Sikhs compared with Muslims living in the same region, specifically in Pakistan.^29^ Within each country, socioeconomic variation also exists and likely influences both health care access and IHD outcomes. Additionally, the absence of rural–urban disaggregation may overlook important within-sex and within-group differences. These unmeasured sources of heterogeneity may confound national-level estimates by masking the experiences of vulnerable subpopulations.

Finally, we were unable to distinguish between different clinical presentations of IHD, such as ST-elevation myocardial infarction, non–ST-elevation myocardial infarction, and unstable or stable angina. This limitation is important because sex disparities in outcomes are more pronounced in ST-elevation myocardial infarction, with women experiencing worse outcomes compared with men, whereas differences may be less significant in other forms of IHD.^30^^,^^31^ Additionally, sex differences in treatment of IHD were not identified and are beyond the scope of this paper, but do impact mortality from IHD and have been documented in South Asia.^32^^,^^33^

## Conclusions

Our study findings indicate that sex disparities in IHD outcomes can vary significantly from country to country, even within the same geographical region. Consistently better outcomes were observed in women compared with men in Bangladesh, Bhutan, India, and Nepal. Conversely, in Pakistan, women experienced the worst outcomes. Potential contributing factors to this disparity include high systolic blood pressure and inadequate intake of vegetables and fiber.

Although blood pressure screening and low-cost antihypertensive medications are becoming increasingly accessible in South Asia, the main challenge in reducing excess IHD mortality among women lies in prevention rather than solely improving hypertension treatment strategies. Promoting a healthy diet that replaces meat and saturated fats with vegetables and other fiber-rich foods could offer substantial health benefits and help to reduce sex disparities in IHD mortality. Efforts to address nutrition-related IHD risks in these populations must be planned both within their countries of origin and in countries where they have migrated.

## Funding Support and Author Disclosures

Dr Gale received grants from Alan Turing Institute, British Heart Foundation, National Institute for Health Research, Horizin 2020, Abbott Diabetes, Bristol Myers Squibb, ESC; consulting fees from AI Nexus, AstraZeneca, Amgen, Bayer, Bristol Myers Squibb, Boehrningher-Ingleheim, CardioMatics, Chiesi, Daiichi-Sankyo, GPRI Research BV, Menarini, Novartis, iRhythm, Organon, the Phoenix Group; Honoraria by AstraZeneca, Boston Scientific, Menarini, Novartis, Raisio Group, Wondr Medical, Zydus; Dr Gale also participated on the data safety monitoring boards of DANBLOCK trial and TARGET CTCA trial, is Chair of the ESC Quality Indicator Committee and was part of the NICE indicator advisory committee; Dr Gulati participated on a data safety monitoring board for Merck, and is Past President of the American Society for Preventive Cardiology. All other authors have reported that they have no relationships relevant to the contents of this paper to disclose.
