# Supplementary material for: Sex Disparities in Ischemic Heart Disease in South Asia: The Role of Dietary Factors
Source: JACC Asia. 2025 Oct 16;6(2):161–74. doi: 10.1016/j.jacasi.2025.07.012 (PMC12904826; doi:10.1016/j.jacasi.2025.07.012)
Supplement: Supplemental Material [file mmc1.docx]

**Appendix -****Supplementary Data-**

**Sex Disparities in Ischemic Heart Disease in South Asia**

**The Role of Dietary Factors**

***Table of Contents***

[Supplemental Figure 1. Pearson correlation analyses: correlation between the ASMR-to-ASPR index and GNI per Capita. Combined data of both men and women. 9](#_Toc201164221)

[Supplemental Figure 2: Pearson correlation analyses: correlation between the ASMR-to-ASPR women-to-men ratios and GNI per Capita. 10](#_Toc201164224)

[Supplemental Figure 3. Pearson correlation coefficients between GNI per capita and the ASMR-to-ASPR index (2005-2021). 11](#_Toc201164227)

[Supplemental Table 1: GNI per Capita (USD) of South Asian countries, 2005 and 2021. GNI per Capita (USD) in 2005 and 2021, by country. Data from World Bank Databank. 12](#_Toc201164228)

[Supplemental Table 2: Dietary risk factor exposure GBD definitions and optimal level of exposure as defined by GBD 2021. 13](#_Toc201164229)

[Supplemental Table 3: 2005-2021 age-standardised prevalence rate, mortality rate, ASMR-to-ASPR index, and risk ratio women to men in individuals of all ages, per 100,000 inhabitants. Data from GBD 2021. 15](#_Toc201164230)

[Supplemental Table 4: Z-Values of ASMR-to-ASPR ratio (95% UI) in 2005 and 2021 in individuals of all ages, per 100,000 inhabitants. Stratified for sex and country. 17](#_Toc201164231)

[Supplemental Table 5: Age-standardised mortality rates (95% UI) for IHD attributable to high LDL cholesterol values. 2021. 18](#_Toc201164232)

[Supplemental Table 6: Age-standardised mortality rates (95% UI) for IHD attributable to high systolic pressure. 2021. 19](#_Toc201164233)

[Supplemental Table 7: Age-standardised mortality rates (95% UI) for IHD attributable to tobacco use. 2021. 20](#_Toc201164234)

[Supplemental Table 8: Age-standardised mortality rates (95% UI) for IHD attributable to elevated body mass index. 2021 21](#_Toc201164235)

[Supplemental Table 9: Age-standardised mortality rates (95% UI) for IHD attributable to elevated fasting plasma glucose. 2021. 22](#_Toc201164236)

[Supplemental Table 10: Age-standardised mortality rates (95% UI) for IHD attributable to air pollution or physical inactivity. 2021. 23](#_Toc201164237)

[Supplemental Table 11: Z-Values of ASMR-to-ASPR ratio (95% UI) for age-standardised mortality rates for IHD attributable to high LDL cholesterol values. 2021. 24](#_Toc201164238)

[Supplemental Table 12: Z-Values of ASMR-to-ASPR ratio (95% UI) for age-standardised mortality rates for IHD attributable to high systolic pressure. 2021. 25](#_Toc201164239)

[Supplemental Table 13: Z-Values of ASMR-to-ASPR ratio (95% UI) for age-standardised mortality rates for IHD attributable to tobacco use. 2021. 26](#_Toc201164240)

[Supplemental Table 14: Z-Values of ASMR-to-ASPR ratio (95% UI) for age-standardised mortality rates for IHD attributable to elevated BMI. 2021. 27](#_Toc201164241)

[Supplemental Table 15: Z-Values of ASMR-to-ASPR ratio (95% UI) for age-standardised mortality rates for IHD attributable to elevated fasting blood plasma glucose. 2021. 28](#_Toc201164242)

[Supplemental Table 16: Z-Values of ASMR-to-ASPR ratio (95% UI) for age-standardised mortality rates for IHD attributable to air pollution. 2021. 29](#_Toc201164243)

[Supplemental Table 17: Z-Values of ASMR-to-ASPR ratio (95% UI) for age-standardised mortality rates for IHD attributable to physical inactivity. 2021. 30](#_Toc201164244)

[Supplemental Table 18. Age-standardised mortality rates (95% UI) for IHD attributable to Diet low in whole grains, 2021. 31](#_Toc201164245)

[Supplemental Table 19. Age-standardised mortality rates (95% UI) for IHD attributable to Diet low in vegetables, 2021. 32](#_Toc201164246)

[Supplemental Table 20. Age-standardised mortality rates (95% UI) for IHD attributable to Diet low in seafood omega-3 fatty acids, 2021. 33](#_Toc201164247)

[Supplemental Table 21. Age-standardised mortality rates (95% UI) for IHD attributable to Diet low in omega-6 polyunsaturated fatty acids, 2021. 34](#_Toc201164248)

[Supplemental Table 22. Age-standardised mortality rates (95% UI) for IHD attributable to Diet low in nuts and seeds, 2021. 35](#_Toc201164249)

[Supplemental Table 23. Age-standardised mortality rates (95% UI) for IHD attributable to Diet low in legumes, 2021. 36](#_Toc201164250)

[Supplemental Table 24. Age-standardised mortality rates (95% UI) for IHD attributable to Diet low in fruits, 2021. 37](#_Toc201164251)

[Supplemental Table 25. Age-standardised mortality rates (95% UI) for IHD attributable to Diet low in fibre, 2021. 38](#_Toc201164252)

[Supplemental Table 26. Age-standardised mortality rates (95% UI) for IHD attributable to Diet high in trans fatty acids, 2021. 39](#_Toc201164253)

[Supplemental Table 27. Age-standardised mortality rates (95% UI) for IHD attributable to Diet high in sugar-sweetened beverages, 2021. 40](#_Toc201164254)

[Supplemental Table 28. Age-standardised mortality rates (95% UI) for IHD attributable to Diet high in sodium, 2021. 41](#_Toc201164255)

[Supplemental Table 29. Age-standardised mortality rates (95% UI) for IHD attributable to Diet high in red meat, 2021. 42](#_Toc201164256)

[Supplemental Table 30. Age-standardised mortality rates (95% UI) for IHD attributable to Diet high in processed meat, 2021. 43](#_Toc201164257)

[Supplemental Table 31: Population* in South Asia in 2005 and 2021, by sex, country. Data from World Bank. 44](#_Toc201164258)

[Supplemental Table 32: Women to men ratios of ASMR-to-ASPR index (95% UI) in 2005, 2010, 2015, 2021 in individuals of all ages, per 100,000 inhabitants. Stratified for sex and country. Data from GBD 2021. 45](#_Toc201164259)

[Supplemental Table 33: GNI per Capita (USD) of South Asian countries, 2005 to 2021. GNI per Capita (USD) in 2005 and 2021, by country. Data from World Bank Databank. 46](#_Toc201164260)

[Supplemental Table 34: Age-standardised prevalence rate of IHD per 100,000 inhabitants (95% UI) 47](#_Toc201164261)

[Supplemental Table 35: Age-standardised prevalence rate of IHD per 100,000 inhabitants (95% UI), continued 48](#_Toc201164262)

[Supplemental Table 36: Age-standardised mortality rate of IHD per 100,000 inhabitants (95% UI) 49](#_Toc201164263)

[Supplemental Table 37: Age-standardised mortality rate of IHD per 100,000 inhabitants (95% UI), continued 50](#_Toc201164264)

[Supplemental Table 38: ASMR-to-ASPR index % (95% UI) 51](#_Toc201164265)

[Supplemental Table 39: ASMR-to-ASPR index % (95% UI), continued 52](#_Toc201164266)

[Supplemental Table 40: Women to men ratio (95% UI) 53](#_Toc201164267)

[Detailed methodology and data sources: GATHER Statement^1^ 54](#_Toc201164268)

[References 56](#_Toc201164269)

**APPENDIX**

**GBD Overview**

The Global Burden of Disease (GBD) Study 2021 provides a comprehensive evaluation of disease burden, covering 371 diseases across 204 countries and territories from 1990 to 2021. It generates various metrics, including prevalence rates and cause-specific mortality, adhering to the Guidelines for Accurate and Transparent Health Estimates Reporting (GATHER).^1^

To expand available data and produce outcomes for the global population, GBD employs a range of modelling tools. This modelling is conducted at the 1000 draw level, with point estimates calculated as the mean of these draws, and 95% uncertainty intervals (UIs) derived from the 2.5th and 97.5th percentiles.^2^

Using the GBD 2021 global population age standard, age-standardized rates are calculated to facilitate comparisons across different locations and time periods with varying age distributions.^3,4^ In countries with robust vital registration systems characterized by high completeness and minimal errors, vital registration serves as the primary data source for causes of death. The majority of causes of death data is obtained from vital registration sources, including those submitted to the World Health Organization (WHO) Mortality Database, as well as country-specific mortality databases managed by national authorities.

**Methods**

**1. Calculation of the ASMR-to-ASPR Index:**

The Age-standardized Mortality Rate (ASMR) and Age-standardized Prevalence Rate (ASPR) were used to evaluate the burden of ischemic heart disease (IHD) relative to its prevalence. ASMR represents IHD-related deaths per 100,000 inhabitants, while ASPR represents individuals diagnosed with IHD per 100,000 inhabitants. Both measures are age-standardized to facilitate cross-population comparisons. The uncertainty intervals for ASMRs and ASPRs are derived from the GBD 2021 dataset, which provides 95% uncertainty bounds based on a combination of data sources and statistical modelling.^5^

**Steps for Calculating the ASMR-to-ASPR Index:**

**(a)** **Point Estimate:** The ASMR-to-ASPR Index was calculated using the following formula:

ASMR-to-ASPR Index=ASMR/ ASPR​.

The point estimate is the ratio of mean ASMR and ASPR estimates.

**(b) Uncertainty Ranges:** To account for uncertainty in the data provided by the GBD study, we calculated the **lower** and **upper bounds** of the ASMR-to-ASPR Index:

***Lower bound of the index*** = Lower uncertainty limit of ASMR/Upper uncertainty limit of ASPR. (assuming the highest prevalence and lowest mortality).

***Upper bound of the index*** = Upper uncertainty limit of ASMR/ Lower uncertainty limit of ASPR. (assuming the lowest prevalence and highest mortality).

These bounds incorporate the 95% uncertainty intervals (UI) provided for ASMR and ASPR.

**(c)** **Expressing the ASMR-to-ASPR Index as a Percentage:** To simplify interpretation, the ASMR-to-ASPR index was expressed as a percentage by multiplying the index by 100. Assuming the following GBD values (fictional for illustration) of ASMR = 150 (95% UI: 130–170) and ASPR = 2000 (95% UI: 1800–2200), the point estimate would be: 0.075, the lower bound would be: 0.059 and the upper bound would be: 0.094. When expressed as a percentage. the ASMR-to-ASPR Index would be: 7.5% (range: 5.9% to 9.4%). This indicates that approximately 7.5% of individuals diagnosed with IHD died from the disease, with an uncertainty range of 5.9% to 9.4%.

**2. Women-to-Men Ratios of the ASMR-to-ASPR Index:**

We calculated the women-to-men risk ratio of the ASMR-to-ASPR index using the following formula:

**(a)** **Point Estimate:** Women-to-Men Risk Ratio=ASMR-to-ASPR Index for Women/ASMR-to-ASPR Index for Men

**(b) Uncertainty Bound Calculations:**

The upper and lower bounds of the women-to-men risk ratio were derived from the upper and lower bounds of the ASMR-to-ASPR ratios for women and men:

***Lower bound of the women-to-men risk ratio:*** =Lower ASMR-to-ASPR Ratio for Women/ Upper ASMR-to-ASPR Ratio for Men

***Upper bound*** ***of the women-to-men risk ratio***: = Upper ASMR-to-ASPR Ratio for Women/ Lower ASMR-to-ASPR Ratio for Men.

These calculations incorporate the uncertainty intervals associated with each sex-specific ratio of the ASMR-to-ASPR index.

**3- Two Proportion Z-Test for Cross-Population Comparisons**

We used a two-proportion Z-test to assess disparities in IHD mortality burden across countries. The ASMR-to-ASPR index for women and men was separately compared against a reference country, Bhutan, the country with the lowest ASMR in both sexes, which served as the reference for Z-score calculations. A Z-score greater than 2.58 (99% confidence level) or greater than 1.96 (95% confidence level) indicated that the ASMR-to-ASPR index in a given country was significantly different from that of the reference country. The same methodology was applied to assess differences in PAF ratios for each risk factor.

4- **Statistical Significance for Sex Differences**

Statistical significance for sex differences was determined indirectly. If the Z-score was significant for women but not for men, this indicated a higher mortality burden in women relative to men in that country. If the Z-score was significant for men but not for women, this indicated a higher mortality burden in men relative to women. If the Z-score was significant for both sexes or neither, no clear sex-specific disparity could be determined. This approach provided a precise and statistically robust assessment of sex disparities in IHD mortality burden across countries, avoiding the issue of excessive uncertainty propagation that arises from comparing women-to-men compounded risk ratios of the ASMR-to-ASPR index.

**5.** **The Z-score was calculated using the following formula:**

$$Z=\frac{(RR_{1}-RR_{2})}{\surd(S{E_{1}}^{2}+S{E_{2}}^{2})}$$

Where:

- RR_1_​ and RR_2_ are the ASMR-to-ASPR index in the two different populations.
- SE1​ and SE2 are the standard errors for the ASMR-to-ASPR index in the two different populations.

A Z-score greater than 1.96 or less than -1.96 was considered statistically significant, corresponding to a 95% confidence level.

**Standard Error Calculation for Risk Ratios**

The standard error (SE) for each risk ratio was calculated to account for the variability within each population’s ratio:

$$SE = \frac{UI Upper-UILower}{1.96\times2}$$

Where: UI Upper and UI lower represent the upper and lower bounds of the 95% uncertainty interval provided by the GBD. This formula derives SE directly from the width of the 95% uncertainty interval.

The standard error of the two ratios was calculated using the formula for the standard error of a ratio, considering the variances of the individual ASMR and ASPR values for each country.

**Example Application:**

To illustrate this methodology, we applied the two-proportion Z-test to compare the ASMR-to-ASPR index (%) in women between Bhutan and Pakistan in 2005:

$$SE = \frac{UI Upper-UILower}{1.96\times2}$$

***Step-by-Step Calculation***

***Step 1: Extract Given Values***

- Bhutan: RR1=2.61 with UI (1.72 – 3.73)
- Pakistan: RR2=4.31 with UI (3.20 – 5.88)

***Step 2: Compute Standard Errors (SE)***

- Using the formula:

For Bhutan (women):

SE1 = $\frac{UI Upper-UILower}{1.96\times2}$ = $\frac{3.73-1.72}{1.96\times2}$ = 0.513

For Pakistan (women):

SE2 = $\frac{UI Upper-UILower}{1.96\times2}$ = $\frac{5.88-3.20}{1.96\times2}$ = 0.684

***Step 3: Compute the Z-Score***

Using the Z-score formula:

Z    = $\frac{(RR_{1}-RR_{2})}{\surd(S{E_{1}}^{2}+S{E_{2}}^{2})}$ = $\frac{(2.61-4.31)}{\surd({0.513}^{2}+{0.684}^{2})}$

First, compute the denominator:

$\surd({0.513}^{2}+{0.684}^{2})$ = $\surd(0.263+0.468)$ =   = $\surd0.731$   = 0.855

Now, compute the numerator:

(2.61 – 4.31)  =  –1.70

Finally:

Z  = $\frac{-1.70}{0.855}$ = –1.99

***Interpretation***

The Z-score is **–1.99**, which exceeds the critical value of –**1.96**, indicating that the difference between Bhutan and Pakistan ASMR-to-ASPR index in 2005 is statistically significant at the **95% confidence level**.

**6. Ischemic heart disease (IHD) definition:**

For the GBD 2021 framework, IHD was modelled as the aggregate of discrete sequelae, including acute myocardial infarction and chronic ischemic heart disease.^6^

**Case Definitions**

1. **Acute Myocardial Infarction (MI):** Defined according to the fourth universal definition of MI,^7^ this condition includes:
   - Clinical evidence of myocardial necrosis in a setting consistent with ischemia.
   - Detection of rising or falling cardiac biomarkers, accompanied by at least one of the following: symptoms of ischemia, new electrocardiographic changes (e.g., ST-segment/T-wave alterations), development of pathological Q waves, imaging evidence of new loss of viable myocardium or regional wall motion abnormalities, or the identification of an intracoronary thrombus.
   - Sudden unexplained cardiac death, involving cardiac arrest without evidence of a non-coronary cause.

Unstable angina was also included when reported separately as specified in the fourth universal definition.

1. **Chronic IHD:**
   - **Stable Angina:** Diagnosed based on clinical symptoms, physician assessment, or the use of nitrates for chest pain relief.
   - **Asymptomatic Ischemic Heart Disease:** Includes cases surviving 28 days post-MI but excludes ECG-based estimates due to their limited specificity and sensitivity.

These precise case definitions ensure consistency in modelling and robust estimation of IHD’s burden within the GBD framework, enabling targeted interventions at regional and global level.

# **Supplemental Figure 1. Pearson correlation analyses: correlation between the ASMR-to-ASPR index and GNI per Capita. Combined data of both men and women.**

# **P-Value: 0·03**

# **Pearson Correlation Coefficient (r): -0·77**

**
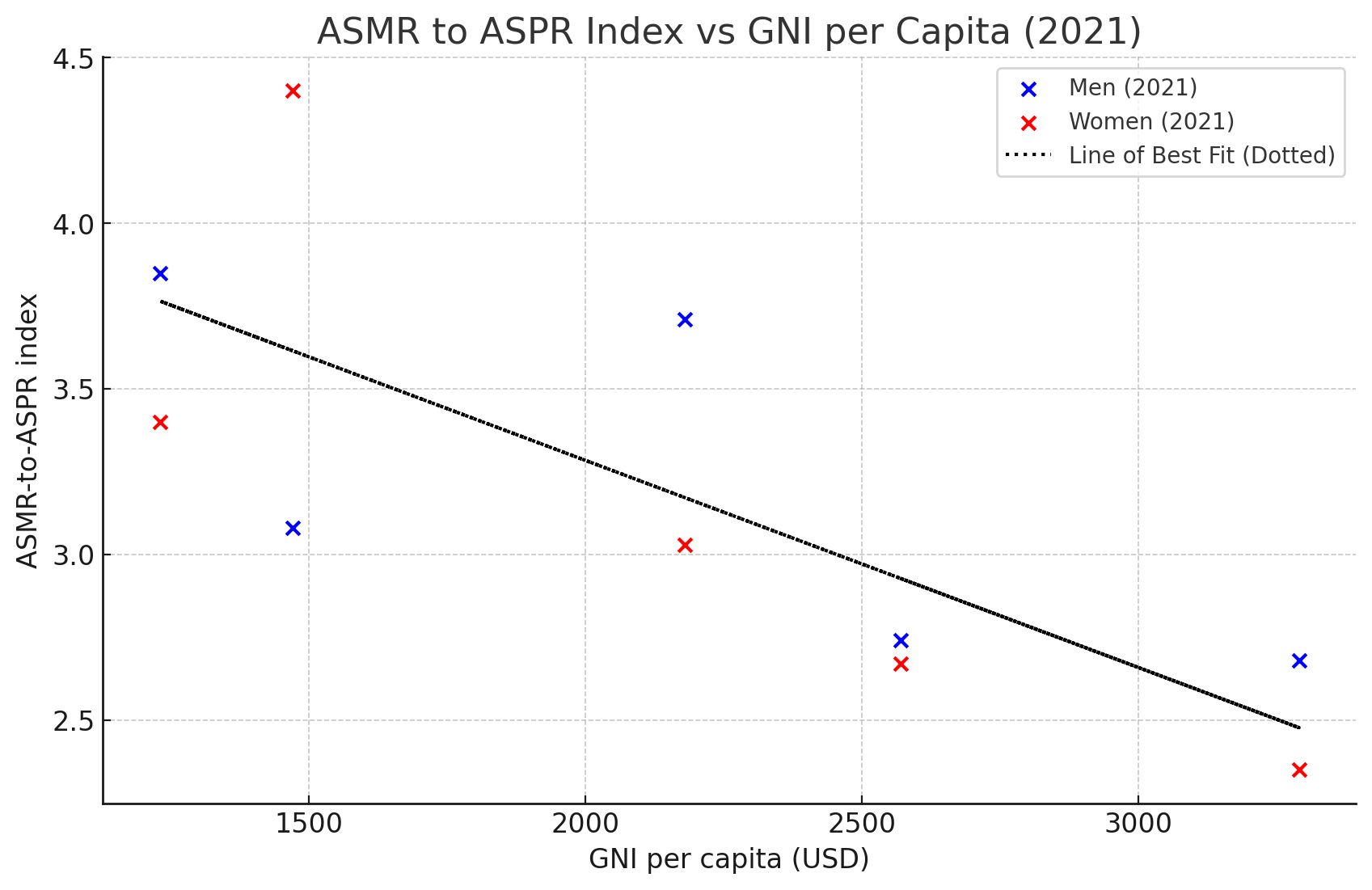
**

# **Supplemental Figure 2: Pearson correlation analyses: correlation between the ASMR-to-ASPR women-to-men ratios and GNI per Capita.**

# **P-Value: 0·36**

# **Pearson Correlation Coefficient (r): -0·33**


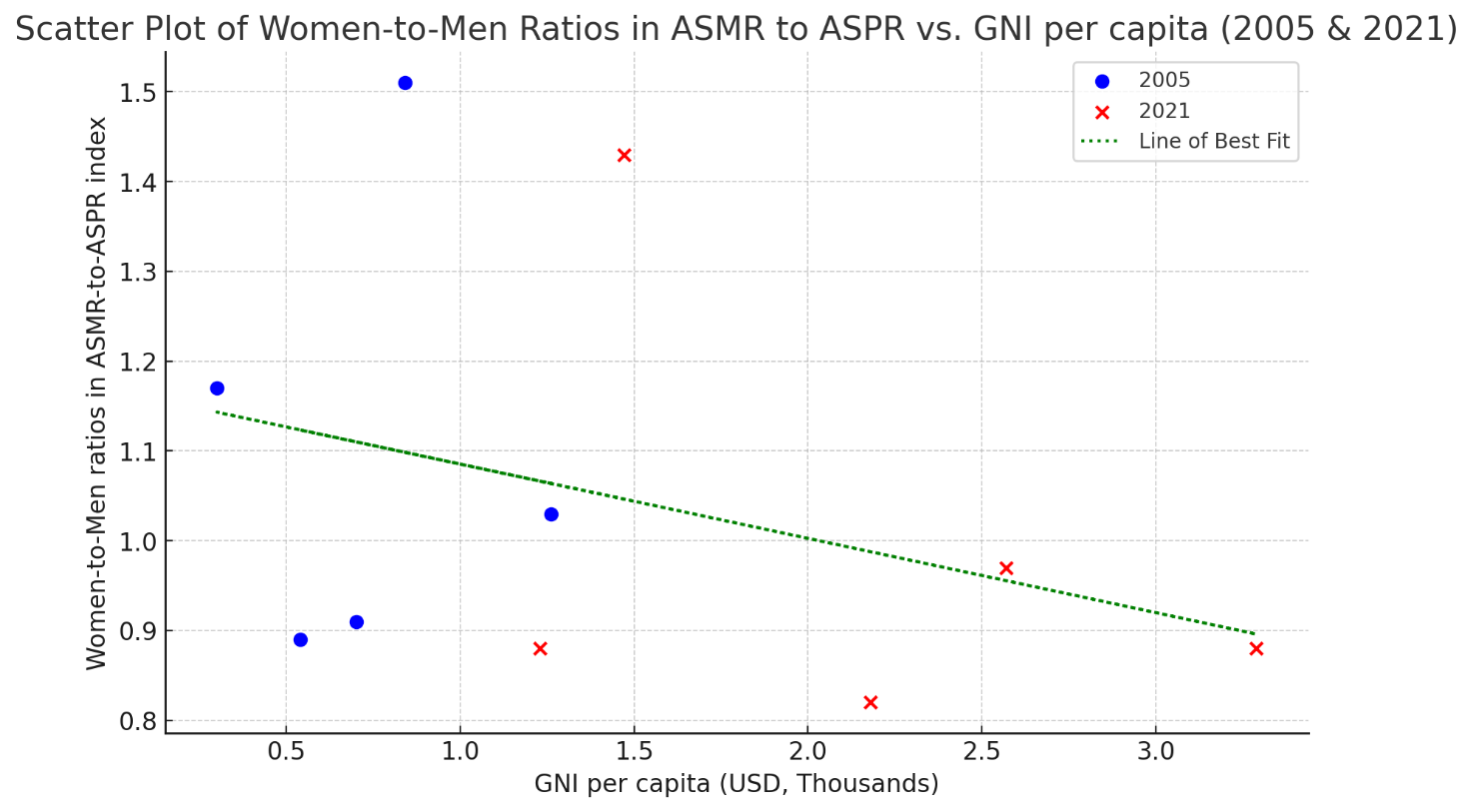


# **Supplemental Figure 3. Pearson correlation coefficients between GNI per capita and the ASMR-to-ASPR index (2005-2021).**


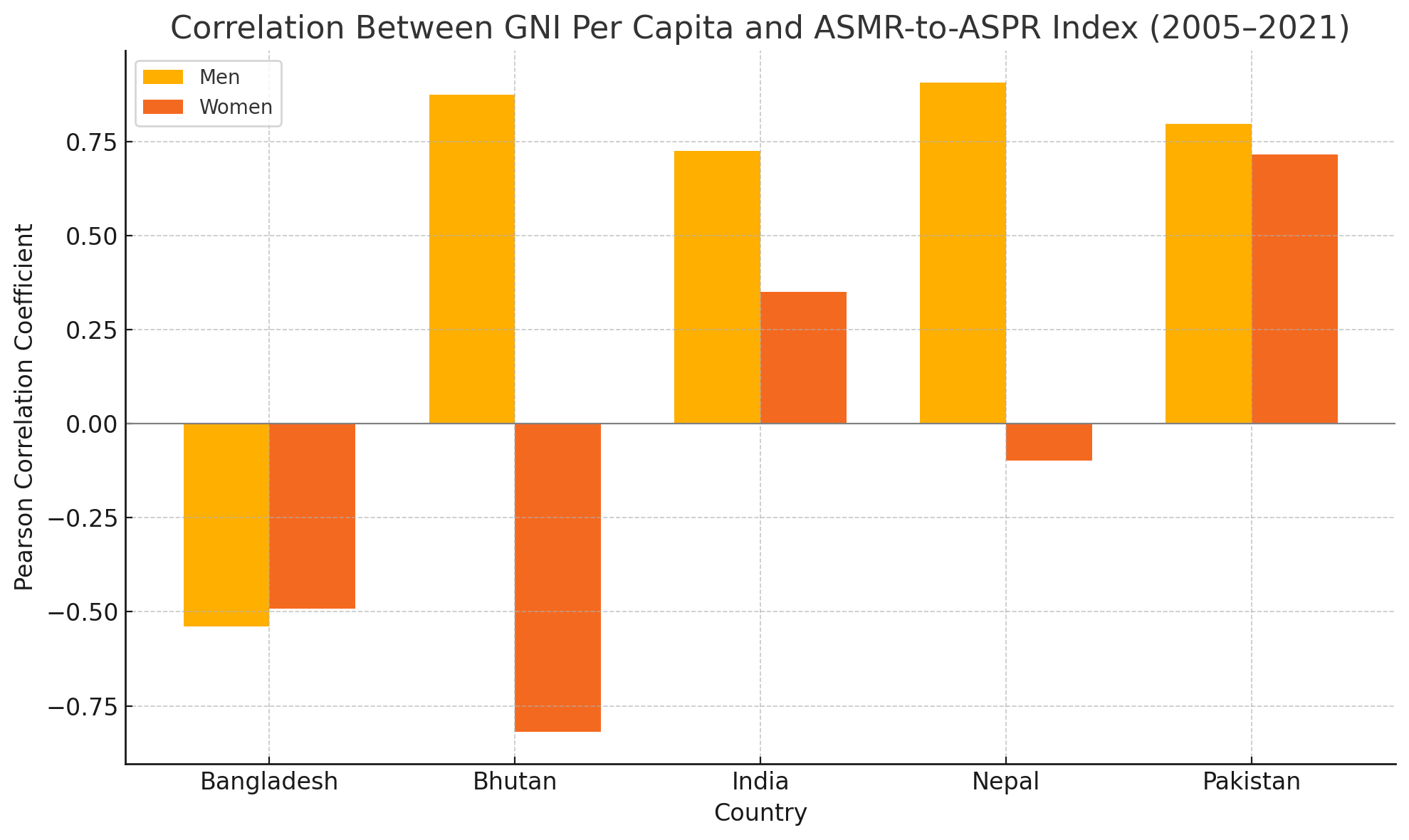


| **Supplemental Table 1: GNI per Capita (USD) of South Asian countries, 2005 and 2021. GNI per Capita (USD) in 2005 and 2021, by country. Data from World Bank Databank.** | | |
| --- | --- | --- |
| **Country** | **2005** | **2021** |
| **Bangladesh** | 540 | 2570 |
| **Bhutan** | 1260 | 3290 |
| **India** | 700 | 2180 |
| **Nepal** | 300 | 1230 |
| **Pakistan** | 840 | 1470 |
| *GNI is based on the de facto definition of population, which counts all residents regardless of legal status or citizenship. The values reported are midyear estimates.  Classifications from 2023-24 World Bank data. South Asian countries classified as low and middle-income (LMICs) with gross domestic product (GNI) per capita of $4,515 or less. | | |
| Data and definition from World bank Databank, Atlas methos (current US$). | | |

| **Supplemental Table 2: Dietary risk factor exposure GBD definitions and optimal level of exposure as defined by GBD 2021.** | | |
| --- | --- | --- |
| **Dietary Risk Factor** | **Definition of Exposure** | **Optimal level or range of intake** |
| **Diet low in fruit** | Average daily consumption (in grams per day) of fruit including fresh, frozen, cooked, canned, or dried fruit, excluding fruit juices and salted or pickled fruits | 340–350 g/day |
| **Diet low in vegetables** | Average daily consumption (in grams per day) of vegetables, including fresh, frozen, cooked, canned, or dried vegetables and excluding legumes and salted or pickled vegetables, juices, nuts and seeds, and starchy vegetables such as potatoes or corn | 306–372 g/day |
| **Diet low in whole grains** | Average daily consumption (in grams per  day) of whole grains (bran, germ, and endosperm in their natural proportion) from breakfast cereals, bread, rice, pasta, biscuits, muffins, tortillas, pancakes, and other sources | 160–210 g/day |
| **Diet low in nuts and seeds** | Average daily consumption (in grams per day) of nuts and seeds, including tree nuts and seeds and peanuts | 19–24 g/day |
| **Diet low in fibre** | Average daily consumption (in grams per day) of fibre from all sources including fruits, vegetables, grains, legumes, and pulses | 22–25 g/day |
| **Diet low in seafood omega-3 fatty acids** | Average daily consumption (in milligrams per day) of eicosapentaenoic acid (EPA) and docosahexaenoic acid (DHA) | 470–660 mg/day |
| **Diet low in omega- 6 polyunsaturated fatty acids** | Average daily consumption (in % daily energy) from omega-6 polyunsaturated fatty acids (PUFA) (specifically linoleic acid, γ- linolenic acid, eicosadienoic acid, dihomo-γ- linolenic acid, arachidonic acid) | 9–10% of total daily energy |
| **Diet low in legumes** | Average daily consumption (in grams per day) of legumes and pulses, including fresh, frozen, cooked, canned, or dried legumes | 100–110 g/day |
| **Diet high in red meat** | Average daily consumption (in grams per day) of unprocessed red meat including pork and bovine meats such as beef, pork, lamb, and goat, but excluding all processed meats, poultry, fish, and eggs | 0–200 g/day |
| **Diet high in processed meat** | Average daily consumption (in grams per day) of meat preserved by smoking, curing, salting, or addition of chemical preservatives | 0 g/day |
| **Diet high in sugar- sweetened beverages (SSBs)** | Average daily consumption (in grams per day) of beverages with ≥50 kcal per 226.8 gram serving, including carbonated beverages, sodas, energy drinks, and fruit drinks, but excluding | 0 g/day |
| **Diet high in trans fatty acids** | Average daily consumption (in percent daily energy) of trans fat from all sources, mainly partially hydrogenated vegetable oils and ruminant products | 0–1.1% of total daily energy |
| **Diet high in sodium** | Average 24-hour urinary sodium excretion (in grams per day) | 1–5 g/day |
| Note that GBD dietary risk factor modelling is for adult populations only (ages 25+). | | |
|  | | |

| **Supplemental Table 3: 2005-2021 age-standardised prevalence rate, mortality rate, ASMR-to-ASPR index, and risk ratio women to men in individuals of all ages, per 100,000 inhabitants. Data from GBD 2021.** | | | | | | | | | | | | | | |
| --- | --- | --- | --- | --- | --- | --- | --- | --- | --- | --- | --- | --- | --- | --- |
| **Country** | **Age-standardised prevalence rate of IHD**  **per 100,000 inhabitants (95% UI)** | | | | **Age-standardized mortality rate for IHD per 100,000 inhabitants (95% UI)** | | | | **ASMR-to-ASPR index**  **% (95% UI)** | | | | **Women to men ratio (95% UI)** | |
|  | **2005** | | **2021** | | **2005** | | **2021** | | **2005** | | **2021** | | **2005** | **2021** |
|  | **Men** | **Women** | **Men** | **Women** | **Men** | **Women** | **Men** | **Women** | **Men** | **Women** | **Men** | **Women** |  |  |
| **Bangladesh** | 4685.91 | 2771.06 | 4876.19 | 3009.09 | 145.17 | 76.10 | 133.61 | 80.41 | 3.10 | 2.75 | 2.74 | 2.67 | 0.89 | 0.97 |
|  | (4318.87 - 5070.59) | (2552.05 - 3006.87) | (4410.08 - 5407.42) | (2682.72 - 3355.03) | (126.54 - 168.35) | (59.88 - 94.99) | (104.20 - 165.80) | (60.94 - 104.61) | (2.50 - 3.90) | (1.99 - 3.72) | (1.93 - 3.76) | (1.82 - 3.90) | (0.51 - 1.49) | (0.48 - 2.02) |
| **Bhutan** | 5188.94 | 3224.97 | 5340.86 | 3366.69 | 132.07 | 84.26 | 143.04 | 79.08 | 2.55 | 2.61 | 2.68 | 2.35 | 1.03 | 0.88 |
|  | (4790.64 - 5653.21) | (2977.46 - 3527.30) | (4768.35 - 5963.44) | (2999.86 - 3791.54) | (95.80 - 170.30) | (60.60 - 111.11) | (109.00 - 178.93) | (55.37 - 107.54) | (1.69 - 3.55) | (1.72 - 3.73) | (1.83 - 3.75) | (1.46 - 3.58) | (0.48 - 2.20) | (0.39 - 1.96) |
| **India** | 5189.58 | 3319.41 | 5322.73 | 3659.84 | 168.68 | 97.84 | 197.53 | 110.77 | 3.25 | 2.95 | 3.71 | 3.03 | 0.91 | 0.82 |
|  | (4538.87 - 5927.17) | (2881.04 - 3798.31) | (4457.39 - 6410.86) | (3094.23 - 4460.36) | (152.90 - 184.64) | (84.07 - 110.02) | (175.08 - 223.59) | (96.09 - 124.77) | (2.58 - 4.07) | (2.21 - 3.82) | (2.73 - 5.02) | (2.15 - 4.03) | (0.54 - 1.48) | (0.43 - 1.48) |
| **Nepal** | 4766.57 | 2929.20 | 4696.58 | 2888.46 | 140.72 | 100.78 | 180.62 | 98.18 | 2.95 | 3.44 | 3.85 | 3.40 | 1.17 | 0.88 |
|  | (4372.47 - 5164.79) | (2700.79 - 3183.39) | (4257.71 - 5196.62) | (2602.81 - 3235.96) | (110.54 - 169.02) | (80.99 - 123.62) | (140.84 - 224.56) | (72.14 - 134.93) | (2.14 - 3.87) | (2.54 - 4.58) | (2.71 - 5.27) | (2.23 - 5.18) | (0.66 - 2.14) | (0.42 - 1.91) |
| **Pakistan** | 6225.66 | 3824.69 | 6274.03 | 3924.14 | 178.04 | 164.96 | 193.50 | 172.65 | 2.86 | 4.31 | 3.08 | 4.40 | 1.51 | 1.43 |
|  | (5473.62 - 7069.97) | (3327.40 - 4381.71) | (5292.03 - 7406.30) | (3307.36 - 4733.61) | (142.62 - 210.96) | (140.00 - 195.59) | (146.39 - 247.52) | (133.43 - 225.97) | (2.02 - 3.85) | (3.20 - 5.88) | (1.98 - 4.68) | (2.82 - 6.83) | (0.83 - 2.91) | (0.60 - 3.46) |
| **South Asia Average** | 5236.14 | 3315.3 | 5347.44 | 3609.07 | 167.12 | 101.63 | 190.07 | 112.14 | 3.19 | 3.07 | 3.55 | 3.11 | 0.96 | 0.87 |
|  | (4617.87 - 5933.63) | (2894.35 - 3777.37) | (4540.29 - 6343.90) | (3083.13 - 4359.80) | (153.08 - 182.18) | (88.74 - 113.45) | (169.87 - 212.11) | (99.08 - 124.35) | (2.58 - 3.95) | (2.35 - 3.92) | (2.68 - 4.67) | (2.27 - 4.03) | (0.60 - 1.52) | (0.49 - 1.51) |
| ASMR= age standardized mortality rate | | | | | | | | | | | | | | |
| ASPR= age standardized prevalence rate | | | | | | | | | | | | | | |
| IHD= ischemic heart disease. GBD 2021 definition describes IHD as International Classification of Disease (ICD) classes I20-25.9, namely: angina pectoris, acute myocardial infarction, subsequent ST elevation (STEMI) and non-ST elevation (NSTEMI) myocardial infarction, certain current complications following ST elevation (STEMI) and non-ST elevation (NSTEMI) myocardial infarction (within the 28-day period), other acute ischemic heart disease, and chronic ischemic heart disease. | | | | | | | | | | | | | | |
| Data and definitions from Global Burden of Disease Database, 2021 | | | | | | | | | | | | | | |

| **Supplemental Table 4: Z-Values of ASMR-to-ASPR ratio (95% UI) in 2005 and 2021 in individuals of all ages, per 100,000 inhabitants. Stratified for sex and country.** | | | | | | | | | | |
| --- | --- | --- | --- | --- | --- | --- | --- | --- | --- | --- |
| **Country** | **ASMR-to-ASPR Men 2005** | **UI Lower Men**  **2005** | **UI Upper Men**  **2005** | **SE Men 2005** | **Z-score Men 2005** | **ASMR-to-ASPR Women 2005** | **UI Lower Women**  **2005** | **UI Upper Women**  **2005** | **SE Women 2005** | **Z-score Women 2005** |
| **Bangladesh** | 3.10 | 2.50 | 3.90 | 0.36 | -0.93 | 2.75 | 1.99 | 3.72 | 0.44 | -0.21 |
| **Bhutan** | 2.55 | 1.69 | 3.55 | 0.47 | 0.00 | 2.61 | 1.72 | 3.73 | 0.51 | 0.00 |
| **India** | 3.25 | 2.58 | 4.07 | 0.38 | -1.15 | 2.95 | 2.21 | 3.82 | 0.41 | -0.52 |
| **Nepal** | 2.95 | 2.14 | 3.87 | 0.44 | -0.62 | 3.44 | 2.54 | 4.58 | 0.52 | -1.14 |
| **Pakistan** | 2.86 | 2.02 | 3.85 | 0.47 | -0.47 | 4.31 | 3.20 | 5.88 | 0.68 | -1.99 |

| **Country** | **ASMR-to-ASPR Men 2021** | **UI Lower Men**  **2021** | **UI Upper Men**  **2021** | **SE Men 2021** | **Z-score Men 2021** | **ASMR-to-ASPR Women 2021** | **UI Lower Women**  **2021** | **UI Upper Women**  **2021** | **SE Women 2021** | **Z-score Women 2021** |
| --- | --- | --- | --- | --- | --- | --- | --- | --- | --- | --- |
| **Bangladesh** | 2.74 | 1.93 | 3.76 | 0.47 | 0.09 | 2.67 | 1.82 | 3.90 | 0.53 | 0.42 |
| **Bhutan** | 2.68 | 1.83 | 3.75 | 0.49 | 0.00 | 2.35 | 1.46 | 3.58 | 0.54 | 0.00 |
| **India** | 3.71 | 2.73 | 5.02 | 0.58 | 1.35 | 3.03 | 2.15 | 4.03 | 0.48 | 0.94 |
| **Nepal** | 3.85 | 2.71 | 5.27 | 0.65 | 1.43 | 3.40 | 2.23 | 5.18 | 0.75 | 1.13 |
| **Pakistan** | 3.08 | 1.98 | 4.68 | 0.69 | 0.47 | 4.40 | 2.82 | 6.83 | 1.02 | 1.77 |

| **Supplemental Table 5: Age-standardised mortality rates (95% UI) for IHD attributable to high LDL cholesterol values. 2021.** | | | |
| --- | --- | --- | --- |
| **Country** | **Men*** | **Women*** | **Women-to-Men ratio** |
| **Bangladesh** | 30.74 | 17.28 | 0.56  (0.22 - 1.36) |
|  | (19.05 - 45.96) | (10.23 - 25.95) |  |
| **Bhutan** | 47.23 | 25.7 | 0.54  (0.23 - 1.41) |
|  | (28.76 - 68.37) | (15.65 - 40.42) |  |
| **India** | 52.54 | 30.76 | 0.59  (0.27 - 1.21) |
|  | (35.04 - 73.75) | (20.24 - 42.57) |  |
| **Nepal** | 45.16 | 25.29 | 0.56  (0.23 - 1.40) |
|  | (27.76 - 65.23) | (14.83 - 38.97) |  |
| **Pakistan** | 55.22 | 49.63 | 0.90  (0.39 - 2.19) |
|  | (33.78 - 81.96) | (32.02 - 74.11) |  |
| Data and definitions from Global Burden of Disease Database. 2021 | | | |
| *****Each value corresponds to mortality rates per 100.000 inhabitants (95% Uncertainty Interval) | | | |

Average Ratio: 0·62

| **Supplemental Table 6: Age-standardised mortality rates (95% UI) for IHD attributable to high systolic pressure. 2021.** | | | |
| --- | --- | --- | --- |
| **Country** | **Men*** | **Women*** | **Women-to-Men ratio** |
| **Bangladesh** | 63.54 | 44.72 | 0.70  (0.37 - 1.32) |
|  | (45.49 - 86.29) | (31.68 - 60.04) |  |
| **Bhutan** | 68.17 | 38.84 | 0.57  (0.27 - 1.16) |
|  | (46.88 - 91.55) | (25.12 - 54.46) |  |
| **India** | 95.24 | 56.98 | 0.60  (0.37 - 0.95) |
|  | (72.72 - 117.95) | (44.09 - 69.18) |  |
| **Nepal** | 78.13 | 29.31 | 0.38  (0.17 - 0.83) |
|  | (55.90 - 105.06) | (17.77 - 46.46) |  |
| **Pakistan** | 100.97 | 95.53 | 0.95  (0.49 - 1.90) |
|  | (66.95 - 135.98) | (66.32 - 126.93) |  |
| Data and definitions from Global Burden of Disease Database. 2021 | | | |
| *****Each value corresponds to mortality rates per 100.000 inhabitants (95% Uncertainty Interval) | | | |

Average Ratio: 0·64

| **Supplemental Table 7: Age-standardised mortality rates (95% UI) for IHD attributable to tobacco use. 2021.** | | | |
| --- | --- | --- | --- |
| **Country** | **Men*** | **Women*** | **Women-to-Men ratio** |
| **Bangladesh** | 40.69 | 6.73 | 0.17  (0.08 - 0.35) |
|  | (29.21 - 53.32) | (4.43 - 10.17) |  |
| **Bhutan** | 22.54 | 4.82 | 0.21  (0.08 - 0.53) |
|  | (14.80 - 34.71) | (2.87 - 7.85) |  |
| **India** | 41.44 | 7.92 | 0.19  (0.11 - 0.31) |
|  | (32.94 - 51.14) | (5.78 - 10.27) |  |
| **Nepal** | 42.62 | 15.49 | 0.36  (0.18 - 0.71) |
|  | (31.30 - 56.21) | (10.17 - 22.10) |  |
| **Pakistan** | 46.98 | 13.16 | 0.28  (0.14 - 0.57) |
|  | (34.19 - 63.01) | (8.75 - 19.37) |  |
| Data and definitions from Global Burden of Disease Database. 2021 | | | |
| *****Each value corresponds to mortality rates per 100.000 inhabitants (95% Uncertainty Interval) | | | |

Average Ratio: 0·24

| **Supplemental Table 8: Age-standardised mortality rates (95% UI) for IHD attributable to elevated body mass index. 2021** | | | |
| --- | --- | --- | --- |
| **Country** | **Men*** | **Women*** | **Women-to-Men ratio** |
| **Bangladesh** | 4.53 | 4.15 | 0.92  (0.18 - 5.01) |
|  | (1.46 - 7.95) | (1.44 - 7.31) |  |
| **Bhutan** | 11.9 | 7.28 | 0.61  (0.12 - 3.12) |
|  | (4.39 - 21.87) | (2.52 - 13.71) |  |
| **India** | 9.74 | 7.24 | 0.74  (0.16 - 3.89) |
|  | (3.52 - 15.89) | (2.52 - 13.71) |  |
| **Nepal** | 8.79 | 3.86 | 0.44  (0.08 - 2.32) |
|  | (3.06 - 15.45) | (1.22 - 7.09) |  |
| **Pakistan** | 14.93 | 16.03 | 1.07  (0.22 - 5.13) |
|  | (5.24 - 27.04) | (6.01 - 26.90) |  |
| Data and definitions from Global Burden of Disease Database. 2021 | | | |
| *****Each value corresponds to mortality rates per 100.000 inhabitants (95% Uncertainty Interval) | | | |

Average Ratio: 0·76

| **Supplemental Table 9: Age-standardised mortality rates (95% UI) for IHD attributable to elevated fasting plasma glucose. 2021.** | | | |
| --- | --- | --- | --- |
| **Country** | **Men*** | **Women*** | **Women-to-Men ratio** |
| **Bangladesh** | 19.43 | 12.22 | 0.63  (0.36 - 1.13) |
|  | (14.51 - 25.04) | (9.07 - 16.34) |  |
| **Bhutan** | 21.28 | 11.41 | 0.54  (0.28 - 1.01) |
|  | (15.70 - 27.93) | (7.92 - 15.84) |  |
| **India** | 32.66 | 17.96 | 0.55  (0.38 - 0.80) |
|  | (26.74 - 39.23) | (14.73 - 21.38) |  |
| **Nepal** | 30.04 | 15.05 | 0.50  (0.27 - 0.87) |
|  | (23.17 - 38.86) | (10.48 - 20.22) |  |
| **Pakistan** | 34.39 | 30.5 | 0.89  (0.50 - 1.54) |
|  | (25.89 - 44.62) | (22.46 - 39.86) |  |
| Data and definitions from Global Burden of Disease Database. 2021 | | | |
| *****Each value corresponds to mortality rates per 100.000 inhabitants (95% Uncertainty Interval) | | | |

Average Ratio: 0·62

| **Supplemental Table 10: Age-standardised mortality rates (95% UI) for IHD attributable to air pollution or physical inactivity. 2021.** | | | | | | |
| --- | --- | --- | --- | --- | --- | --- |
| **Country** | **Air Pollution** | | **Women-to-Men ratio** | **Physical Inactivity** | | **Women-to-Men ratio** |
|  | **Men*** | **Women*** |  | **Men*** | **Women*** |  |
| **Bangladesh** | 59.27 | 35.62 | 0.60  (0.32 - 1.16) | 0.43 | 2.5 | 5.81  (1.01 - 34.92) |
|  | (41.01 - 77.86) | (24.61 - 47.38) |  | (0.13 - 0.94) | (0.95 - 4.54) |  |
| **Bhutan** | 43.48 | 24.5 | 0.56  (0.26 - 1.25) | 6.44 | 6.87 | 1.07  (0.23 - 4.78) |
|  | (28.33 - 58.92) | (15.54 - 35.44) |  | (2.60 - 11.85) | (2.73 - 12.43) |  |
| **India** | 78.07 | 44.01 | 0.56  (0.34 - 0.93) | 4.13 | 5.01 | 1.21  (0.29 - 5.19) |
|  | (59.03 - 97.27) | (33.42 - 54.94) |  | (1.65 - 7.54) | (2.19 - 8.56) |  |
| **Nepal** | 79.72 | 43.37 | 0.54  (0.28 - 1.10) | 1.1 | 1.97 | 1.79  (0.22 - 12.14) |
|  | (56.49 - 104.56) | (28.89 - 61.96) |  | (0.35 - 2.63) | (0.58 - 4.25) |  |
| **Pakistan** | 81.05 | 72.68 | 0.90  (0.47 - 1.74) | 2.61 | 4.55 | 1.74  (0.31 - 9.90) |
|  | (57.03 - 108.37) | (50.69 - 99.51) |  | (0.84 - 5.27) | (1.65 - 8.32) |  |
| Data and definitions from Global Burden of Disease Database. 2021 | | | | | | |
| *****Each value corresponds to mortality rates per 100.000 inhabitants (95% Uncertainty Interval) | | | | | | |

| **Supplemental Table 11: Z-Values of ASMR-to-ASPR ratio (95% UI) for age-standardised mortality rates for IHD attributable to high LDL cholesterol values. 2021.** | | | | | | | | | | |
| --- | --- | --- | --- | --- | --- | --- | --- | --- | --- | --- |
| **Country** | **ASMR Men** | **UI Lower Men** | **UI Upper Men** | **SE Men** | **ASMR Women** | **UI Lower Women** | **UI Upper Women** | **SE Women** | **Z-score Men** | **Z-score Women** |
| **Bhutan** | 47.23 | 28.76 | 68.37 | 10.10 | 25.70 | 15.65 | 40.42 | 6.32 | 0.00 | 0.00 |
| **Bangladesh** | 30.74 | 19.05 | 45.96 | 6.86 | 17.28 | 10.23 | 25.95 | 4.01 | -1.35 | -1.13 |
| **India** | 52.54 | 35.04 | 73.75 | 9.88 | 30.76 | 20.24 | 42.57 | 5.70 | 0.38 | 0.59 |
| **Nepal** | 45.16 | 27.76 | 65.23 | 9.56 | 25.29 | 14.83 | 38.97 | 6.16 | -0.15 | -0.05 |
| **Pakistan** | 55.22 | 33.78 | 81.96 | 12.29 | 49.63 | 32.02 | 74.11 | 10.74 | 0.50 | 1.92 |

| **Supplemental Table 12: Z-Values of ASMR-to-ASPR ratio (95% UI) for age-standardised mortality rates for IHD attributable to high systolic pressure. 2021.** | | | | | | | | | | |
| --- | --- | --- | --- | --- | --- | --- | --- | --- | --- | --- |
| **Country** | **ASMR Men** | **UI Lower Men** | **UI Upper Men** | **SE Men** | **ASMR Women** | **UI Lower Women** | **UI Upper Women** | **SE Women** | **Z-score Men** | **Z-score Women** |
| **Bhutan** | 68.17 | 46.88 | 91.55 | 11.40 | 38.84 | 25.12 | 54.46 | 7.48 | 0.00 | 0.00 |
| **Bangladesh** | 63.54 | 45.49 | 86.29 | 10.41 | 44.72 | 31.68 | 60.04 | 7.23 | -0.30 | 0.56 |
| **India** | 95.24 | 72.72 | 117.95 | 11.54 | 56.98 | 44.09 | 69.18 | 6.40 | 1.67 | 1.84 |
| **Nepal** | 78.13 | 55.90 | 105.06 | 12.54 | 29.31 | 17.77 | 46.46 | 7.32 | 0.59 | -0.91 |
| **Pakistan** | 100.97 | 66.95 | 135.98 | 17.61 | 95.53 | 66.32 | 126.93 | 15.46 | 1.56 | 3.30 |

| **Supplemental Table 13: Z-Values of ASMR-to-ASPR ratio (95% UI) for age-standardised mortality rates for IHD attributable to tobacco use. 2021.** | | | | | | | | | | |
| --- | --- | --- | --- | --- | --- | --- | --- | --- | --- | --- |
| **Country** | **ASMR Men** | **UI Lower Men** | **UI Upper Men** | **SE Men** | **ASMR Women** | **UI Lower Women** | **UI Upper Women** | **SE Women** | **Z-score Men** | **Z-score Women** |
| **Bhutan** | 22.54 | 14.80 | 34.71 | 5.08 | 4.82 | 2.87 | 7.85 | 1.27 | 0.00 | 0.00 |
| **Bangladesh** | 40.69 | 29.21 | 53.32 | 6.15 | 6.73 | 4.43 | 10.17 | 1.46 | 2.28 | 0.99 |
| **India** | 41.44 | 32.94 | 51.14 | 4.64 | 7.92 | 5.78 | 10.27 | 1.15 | 2.75 | 1.81 |
| **Nepal** | 42.62 | 31.30 | 56.21 | 6.35 | 15.49 | 10.17 | 22.10 | 3.04 | 2.47 | 3.24 |
| **Pakistan** | 46.98 | 34.19 | 63.01 | 7.35 | 13.16 | 8.75 | 19.37 | 2.71 | 2.74 | 2.79 |

| **Supplemental Table 14: Z-Values of ASMR-to-ASPR ratio (95% UI) for age-standardised mortality rates for IHD attributable to elevated BMI. 2021.** | | | | | | | | | | |
| --- | --- | --- | --- | --- | --- | --- | --- | --- | --- | --- |
| **Country** | **ASMR Men** | **UI Lower Men** | **UI Upper Men** | **SE Men** | **ASMR Women** | **UI Lower Women** | **UI Upper Women** | **SE Women** | **Z-score Men** | **Z-score Women** |
| **Bhutan** | 11.90 | 4.39 | 21.87 | 4.46 | 7.28 | 2.52 | 13.71 | 2.85 | 0.00 | 0.00 |
| **Bangladesh** | 4.53 | 1.46 | 7.95 | 1.66 | 4.15 | 1.44 | 7.31 | 1.50 | -1.55 | -0.97 |
| **India** | 9.74 | 3.52 | 15.89 | 3.16 | 7.24 | 2.52 | 13.71 | 2.85 | -0.40 | -0.01 |
| **Nepal** | 8.79 | 3.06 | 15.45 | 3.16 | 3.86 | 1.22 | 7.09 | 1.50 | -0.57 | -1.06 |
| **Pakistan** | 14.93 | 5.24 | 27.04 | 5.56 | 16.03 | 6.01 | 26.90 | 5.33 | 0.43 | 1.45 |

| **Supplemental Table 15: Z-Values of ASMR-to-ASPR ratio (95% UI) for age-standardised mortality rates for IHD attributable to elevated fasting blood plasma glucose. 2021.** | | | | | | | | | | |
| --- | --- | --- | --- | --- | --- | --- | --- | --- | --- | --- |
| **Country** | **ASMR Men** | **UI Lower Men** | **UI Upper Men** | **SE Men** | **ASMR Women** | **UI Lower Women** | **UI Upper Women** | **SE Women** | **Z-score Men** | **Z-score Women** |
| **Bhutan** | 21.28 | 15.70 | 27.93 | 3.12 | 11.41 | 7.92 | 15.84 | 2.02 | 0.00 | 0.00 |
| **Bangladesh** | 19.43 | 14.51 | 25.04 | 2.69 | 12.22 | 9.07 | 16.34 | 1.85 | -0.45 | 0.30 |
| **India** | 32.66 | 26.74 | 39.23 | 3.19 | 17.96 | 14.73 | 21.38 | 1.70 | 2.55 | 2.48 |
| **Nepal** | 30.04 | 23.17 | 38.86 | 4.00 | 15.05 | 10.48 | 20.22 | 2.48 | 1.73 | 1.14 |
| **Pakistan** | 34.39 | 25.89 | 44.62 | 4.78 | 30.50 | 22.46 | 39.86 | 4.44 | 2.30 | 3.91 |

| **Supplemental Table 16: Z-Values of ASMR-to-ASPR ratio (95% UI) for age-standardised mortality rates for IHD attributable to air pollution. 2021.** | | | | | | | | | | |
| --- | --- | --- | --- | --- | --- | --- | --- | --- | --- | --- |
| **Country** | **ASMR Men** | **UI Lower Men** | **UI Upper Men** | **SE Men** | **ASMR Women** | **UI Lower Women** | **UI Upper Women** | **SE Women** | **Z-score Men** | **Z-score Women** |
| **Bhutan** | 43.48 | 28.33 | 58.92 | 7.80 | 24.50 | 15.54 | 35.44 | 5.08 | 0.00 | 0.00 |
| **Bangladesh** | 59.27 | 41.01 | 77.86 | 9.40 | 35.62 | 24.61 | 47.38 | 5.81 | 1.29 | 1.44 |
| **India** | 78.07 | 59.03 | 97.27 | 9.76 | 44.01 | 33.42 | 54.94 | 5.49 | 2.77 | 2.61 |
| **Nepal** | 79.72 | 56.49 | 104.56 | 12.26 | 43.37 | 28.89 | 61.96 | 8.44 | 2.49 | 1.92 |
| **Pakistan** | 81.05 | 57.03 | 108.37 | 13.10 | 72.68 | 50.69 | 99.51 | 12.45 | 2.46 | 3.58 |

| **Supplemental Table 17: Z-Values of ASMR-to-ASPR ratio (95% UI) for age-standardised mortality rates for IHD attributable to physical inactivity. 2021.** | | | | | | | | | | |
| --- | --- | --- | --- | --- | --- | --- | --- | --- | --- | --- |
| **Country** | **ASMR Men** | **UI Lower Men** | **UI Upper Men** | **SE Men** | **ASMR Women** | **UI Lower Women** | **UI Upper Women** | **SE Women** | **Z-score Men** | **Z-score Women** |
| **Bhutan** | 6.44 | 2.60 | 11.85 | 2.36 | 6.87 | 2.73 | 12.43 | 2.47 | 0.00 | 0.00 |
| **Bangladesh** | 0.43 | 0.13 | 0.94 | 0.21 | 2.50 | 0.95 | 4.54 | 0.92 | -2.54 | -1.66 |
| **India** | 4.13 | 1.65 | 7.54 | 1.50 | 5.01 | 2.19 | 8.56 | 1.63 | -0.83 | -0.63 |
| **Nepal** | 1.10 | 0.35 | 2.63 | 0.58 | 1.97 | 0.58 | 4.25 | 0.94 | -2.20 | -1.85 |
| **Pakistan** | 2.61 | 0.84 | 5.27 | 1.13 | 4.55 | 1.65 | 8.32 | 1.70 | -1.46 | -0.77 |

| **Supplemental Table 18. Age-standardised mortality rates (95% UI) for IHD attributable to Diet low in whole grains, 2021.** | | | |
| --- | --- | --- | --- |
| **Country** | **Men*** | **Women*** | **Women-to-Men ratio** |
| **Bangladesh** | 8.12 | 3.91 | 0.48  1.37  0.17 |
| UB | 12.00 | 5.87 |  |
| LB | 4.29 | 2.10 |  |
| **Bhutan** | 12.64 | 6.09 | 0.48  1.37  0.15 |
| UB  LB | 19.44 | 9.26 |  |
|  | 6.77 | 2.97 |  |
| **India** | 22.60 | 10.52 | 0.47  1.10  0.21 |
| UB | 30.88 | 14.74 |  |
| LB | 13.38 | 6.33 |  |
| **Nepal** | 15.67 | 7.32 | 0.47  1.33  0.16 |
| UB | 23.52 | 11.08 |  |
| LB | 8.36 | 3.71 |  |
| **Pakistan** | 40.74 | 34.18 | 0.84  2.15  0.31 |
| UB | 61.17 | 50.04 |  |
| LB | 23.25 | 18.90 |  |
| Data and definitions from Global Burden of Disease Database. 2021 | | | |
| *****Each value corresponds to mortality rates per 100.000 inhabitants (95% Uncertainty Interval) | | | |

|  | **SE Men** | **SE Women** | **Z-Score Men** | **Z-Score Women** |
| --- | --- | --- | --- | --- |
| **Bangladesh** | 1.97 | 0.96 | -1.19 | -1.16 |
| **Bhutan** | 3.23 | 1.61 | 0.00 | 0.00 |
| **India** | 4.46 | 2.14 | 1.81 | 1.65 |
| **Nepal** | 3.87 | 1.88 | 0.60 | 0.50 |
| **Pakistan** | 9.67 | 7.94 | 2.76 | 3.47 |

| **Supplemental Table 19. Age-standardised mortality rates (95% UI) for IHD attributable to Diet low in vegetables, 2021.** | | | |
| --- | --- | --- | --- |
| **Country** | **Men*** | **Women*** | **Women-to-Men ratio** |
| **Bangladesh** | 8.77 | 4.68 | 0.53  1.87  0.15 |
| UB | 13.79 | 7.47 |  |
| LB | 3.99 | 2.02 |  |
| **Bhutan** | 5.54 | 2.96 | 0.53  2.03  0.13 |
| UB | 9.10 | 5.04 |  |
| LB | 2.48 | 1.21 |  |
| **India** | 7.53 | 4.20 | 0.56  1.81  0.17 |
| UB | 11.55 | 6.48 |  |
| LB | 3.58 | 1.95 |  |
| **Nepal** | 7.02 | 3.70 | 0.53  1.90  0.13 |
| UB | 11.81 | 6.03 |  |
| LB | 3.17 | 1.52 |  |
| **Pakistan** | 10.39 | 8.57 | 0.82  3.02  0.22 |
| UB | 17.49 | 13.97 |  |
| LB | 4.62 | 3.77 |  |
| Data and definitions from Global Burden of Disease Database. 2021 | | | |
| *****Each value corresponds to mortality rates per 100.000 inhabitants (95% Uncertainty Interval) | | | |

|  | **SE Men** | **SE Women** | **Z-Score Men** | **Z-Score Women** |
| --- | --- | --- | --- | --- |
| **Bangladesh** | 2.50 | 1.39 | 1.07 | 1.01 |
| **Bhutan** | 1.69 | 0.98 | 0.00 | 0.00 |
| **India** | 2.03 | 1.16 | 0.75 | 0.82 |
| **Nepal** | 2.20 | 1.15 | 0.53 | 0.49 |
| **Pakistan** | 3.28 | 2.60 | 1.32 | 2.02 |

| **Supplemental Table 20. Age-standardised mortality rates (95% UI) for IHD attributable to Diet low in seafood omega-3 fatty acids, 2021.** | | | |
| --- | --- | --- | --- |
| **Country** | **Men*** | **Women*** | **Women-to-Men ratio** |
| **Bangladesh** | 2.79 | 3.23 | 1.16  12.43  0.09 |
| UB | 5.81 | 6.24 |  |
| LB | 0.50 | 0.55 |  |
| **Bhutan** | 12.84 | 8.59 | 0.67  7.08  0.07 |
| UB | 23.51 | 15.72 |  |
| LB | 2.22 | 1.56 |  |
| **India** | 18.89 | 12.29 | 0.65  5.65  0.08 |
| UB | 32.27 | 21.10 |  |
| LB | 3.73 | 2.50 |  |
| **Nepal** | 24.49 | 14.01 | 0.57  5.37  0.07 |
| UB | 43.31 | 24.86 |  |
| LB | 4.63 | 2.93 |  |
| **Pakistan** | 26.15 | 24.26 | 0.93  7.62  0.10 |
| UB | 48.29 | 42.69 |  |
| LB | 5.60 | 4.85 |  |
| Data and definitions from Global Burden of Disease Database. 2021 | | | |
| *****Each value corresponds to mortality rates per 100.000 inhabitants (95% Uncertainty Interval) | | | |

|  | **SE Men** | **SE Women** | **Z-Score Men** | **Z-Score Women** |
| --- | --- | --- | --- | --- |
| **Bangladesh** | 1.35 | 1.45 | -1.80 | -1.38 |
| **Bhutan** | 5.43 | 3.61 | 0.00 | 0.00 |
| **India** | 7.28 | 4.74 | 0.67 | 0.62 |
| **Nepal** | 9.87 | 5.59 | 1.03 | 0.81 |
| **Pakistan** | 10.89 | 9.65 | 1.09 | 1.52 |

| **Supplemental Table 21. Age-standardised mortality rates (95% UI) for IHD attributable to Diet low in omega-6 polyunsaturated fatty acids, 2021.** | | | |
| --- | --- | --- | --- |
| **Country** | **Men*** | **Women*** | **Women-to-Men ratio** |
| **Bangladesh** | 12.85 | 7.17 | 0.56  -0.71  -0.43 |
| UB | 48.81 | 27.45 |  |
| LB | -38.83 | -20.94 |  |
| **Bhutan** | 12.97 | 7.10 | 0.55  -0.72  -0.36 |
| UB | 50.53 | 27.16 |  |
| LB | -37.49 | -18.10 |  |
| **India** | 19.32 | 10.44 | 0.54  -0.68  -0.43 |
| UB | 72.85 | 39.88 |  |
| LB | -58.59 | -31.41 |  |
| **Nepal** | 14.57 | 7.85 | 0.54  -0.78  -0.36 |
| UB | 56.28 | 29.98 |  |
| LB | -38.54 | -20.38 |  |
| **Pakistan** | 19.24 | 16.34 | 0.85  -1.13  -0.59 |
| UB | 77.26 | 64.98 |  |
| LB | -57.28 | -45.80 |  |
| Data and definitions from Global Burden of Disease Database. 2021 | | | |
| *****Each value corresponds to mortality rates per 100.000 inhabitants (95% Uncertainty Interval). Negative values in the UI indicate overlapping intervals for men and women. | | | |

|  | **SE Men** | **SE Women** | **Z-Score Men** | **Z-Score Women** |
| --- | --- | --- | --- | --- |
| **Bangladesh** | 22.36 | 12.34 | 0.00 | 0.00 |
| **Bhutan** | 22.45 | 11.55 | 0.00 | 0.00 |
| **India** | 33.53 | 18.18 | 0.16 | 0.15 |
| **Nepal** | 24.19 | 12.85 | 0.05 | 0.04 |
| **Pakistan** | 34.32 | 28.26 | 0.15 | 0.30 |

| **Supplemental Table 22. Age-standardised mortality rates (95% UI) for IHD attributable to Diet low in nuts and seeds, 2021.** | | | |
| --- | --- | --- | --- |
| **Country** | **Men*** | **Women*** | **Women-to-Men ratio** |
| **Bangladesh** | 19.62 | 10.55 | 0.54  3.27  0.07 |
| UB | 33.51 | 17.98 |  |
| LB | 5.50 | 2.51 |  |
| **Bhutan** | 12.61 | 6.21 | 0.49  3.50  0.07 |
| UB | 22.85 | 11.43 |  |
| LB | 3.26 | 1.51 |  |
| **India** | 20.59 | 10.80 | 0.52  3.04  0.09 |
| UB | 33.28 | 17.88 |  |
| LB | 5.87 | 3.06 |  |
| **Nepal** | 23.03 | 11.63 | 0.51  3.43  0.08 |
| UB | 39.29 | 20.00 |  |
| LB | 5.83 | 3.30 |  |
| **Pakistan** | 21.38 | 16.98 | 0.79  4.71  0.12 |
| UB | 37.79 | 29.23 |  |
| LB | 6.20 | 4.62 |  |
| Data and definitions from Global Burden of Disease Database. 2021 | | | |
| *****Each value corresponds to mortality rates per 100.000 inhabitants (95% Uncertainty Interval). | | | |

|  | **SE Men** | **SE Women** | **Z-Score Men** | **Z-Score Women** |
| --- | --- | --- | --- | --- |
| **Bangladesh** | 7.15 | 3.95 | 0.80 | 0.92 |
| **Bhutan** | 5.00 | 2.53 | 0.00 | 0.00 |
| **India** | 6.99 | 3.78 | 0.93 | 1.01 |
| **Nepal** | 8.54 | 4.26 | 1.05 | 1.09 |
| **Pakistan** | 8.06 | 6.28 | 0.93 | 1.59 |

| **Supplemental Table 23. Age-standardised mortality rates (95% UI) for IHD attributable to Diet low in legumes, 2021.** | | | |
| --- | --- | --- | --- |
| **Country** | **Men*** | **Women*** | **Women-to-Men ratio** |
| **Bangladesh** | 7.26 | 3.66 | 0.50  -1.76  -0.15 |
| UB | 19.46 | 9.79 |  |
| LB | -5.56 | -2.83 |  |
| **Bhutan** | 4.30 | 2.03 | 0.47  -1.71  -0.12 |
| UB | 11.50 | 5.66 |  |
| LB | -3.31 | -1.43 |  |
| **India** | 7.39 | 4.43 | 0.60  --2.00  -0.19 |
| UB | 18.95 | 11.52 |  |
| LB | -5.75 | -3.52 |  |
| **Nepal** | 7.26 | 3.46 | 0.48  -1.78  -0.13 |
| UB | 19.49 | 9.74 |  |
| LB | -5.49 | -2.48 |  |
| **Pakistan** | 9.63 | 7.42 | 0.77  -2.70  -0.21 |
| UB | 26.73 | 20.15 |  |
| LB | -7.45 | -5.58 |  |
| Data and definitions from Global Burden of Disease Database. 2021 | | | |
| *****Each value corresponds to mortality rates per 100.000 inhabitants (95% Uncertainty Interval). Negative values in the UI indicate overlapping intervals for men and women. | | | |

|  | **SE Men** | **SE Women** | **Z-Score Men** | **Z-Score Women** |
| --- | --- | --- | --- | --- |
| **Bangladesh** | 6.38 | 3.22 | 0.40 | 0.44 |
| **Bhutan** | 3.78 | 1.81 | 0.00 | 0.00 |
| **India** | 6.30 | 3.84 | 0.42 | 0.57 |
| **Nepal** | 6.37 | 3.12 | 0.40 | 0.40 |
| **Pakistan** | 8.72 | 6.56 | 0.56 | 0.79 |

| **Supplemental Table 24. Age-standardised mortality rates (95% UI) for IHD attributable to Diet low in fruits, 2021.** | | | |
| --- | --- | --- | --- |
| **Country** | **Men*** | **Women*** | **Women-to-Men ratio** |
| **Bangladesh** | 16.84 | 9.31 | 0.55  4.69  0.06 |
| UB | 29.57 | 16.22 |  |
| LB | 3.46 | 1.69 |  |
| **Bhutan** | 11.77 | 6.50 | 0.55  5.26  0.05 |
| UB | 21.45 | 12.25 |  |
| LB | 2.33 | 1.17 |  |
| **India** | 28.04 | 15.22 | 0.54  4.24  0.07 |
| UB | 46.24 | 25.68 |  |
| LB | 6.06 | 3.20 |  |
| **Nepal** | 14.73 | 7.93 | 0.54  4.94  0.06 |
| UB | 26.90 | 14.17 |  |
| LB | 2.87 | 1.55 |  |
| **Pakistan** | 18.84 | 16.10 | 0.85  7.02  0.09 |
| UB | 34.57 | 29.02 |  |
| LB | 4.14 | 3.25 |  |
| Data and definitions from Global Burden of Disease Database. 2021 | | | |
| *****Each value corresponds to mortality rates per 100.000 inhabitants (95% Uncertainty Interval). | | | |

|  | **SE Men** | **SE Women** | **Z-Score Men** | **Z-Score Women** |
| --- | --- | --- | --- | --- |
| **Bangladesh** | 6.66 | 3.71 | 0.61 | 0.60 |
| **Bhutan** | 4.88 | 2.83 | 0.00 | 0.00 |
| **India** | 10.25 | 5.73 | 1.43 | 1.36 |
| **Nepal** | 6.13 | 3.22 | 0.38 | 0.33 |
| **Pakistan** | 7.76 | 6.57 | 0.77 | 1.34 |

| **Supplemental Table 25. Age-standardised mortality rates (95% UI) for IHD attributable to Diet low in fibre, 2021.** | | | |
| --- | --- | --- | --- |
| **Country** | **Men*** | **Women*** | **Women-to-Men ratio** |
| **Bangladesh** | 23.12 | 12.80 | 0.55  1.71  0.18 |
| UB | 35.19 | 19.27 |  |
| LB | 11.30 | 6.21 |  |
| **Bhutan** | 12.10 | 6.62 | 0.55  1.84  0.15 |
| UB | 19.75 | 10.74 |  |
| LB | 5.83 | 2.96 |  |
| **India** | 10.57 | 5.80 | 0.55  1.63  0.18 |
| UB | 15.97 | 8.84 |  |
| LB | 5.41 | 2.94 |  |
| **Nepal** | 8.34 | 4.53 | 0.54  2.10  0.14 |
| UB | 13.54 | 7.76 |  |
| LB | 3.70 | 1.96 |  |
| **Pakistan** | 19.77 | 16.82 | 0.85  2.73  0.26 |
| UB | 31.33 | 26.52 |  |
| LB | 9.71 | 8.13 |  |
| Data and definitions from Global Burden of Disease Database. 2021 | | | |
| *****Each value corresponds to mortality rates per 100.000 inhabitants (95% Uncertainty Interval). | | | |

|  | **SE Men** | **SE Women** | **Z-Score Men** | **Z-Score Women** |
| --- | --- | --- | --- | --- |
| **Bangladesh** | 6.10 | 3.33 | 1.56 | 1.59 |
| **Bhutan** | 3.55 | 1.98 | 0.00 | 0.00 |
| **India** | 2.69 | 1.51 | -0.34 | -0.33 |
| **Nepal** | 2.51 | 1.48 | -0.87 | -0.84 |
| **Pakistan** | 5.52 | 4.69 | 1.17 | 2.00 |

| **Supplemental Table 26. Age-standardised mortality rates (95% UI) for IHD attributable to Diet high in trans fatty acids, 2021.** | | | |
| --- | --- | --- | --- |
| **Country** | **Men*** | **Women*** | **Women-to-Men ratio** |
| **Bangladesh** | 3.44 | 1.92 | 0.56  13.28  0.03 |
| UB | 7.22 | 4.04 |  |
| LB | 0.30 | 0.19 |  |
| **Bhutan** | 3.47 | 1.90 | 0.55  13.58  0.02 |
| UB | 7.47 | 3.95 |  |
| LB | 0.29 | 0.13 |  |
| **India** | 4.94 | 2.63 | 0.53  11.94  0.03 |
| UB | 9.70 | 5.65 |  |
| LB | 0.47 | 0.28 |  |
| **Nepal** | 4.44 | 2.35 | 0.53  12.16  0.02 |
| UB | 9.28 | 4.81 |  |
| LB | 0.40 | 0.23 |  |
| **Pakistan** | 5.12 | 4.39 | 0.86  21.01  0.04 |
| UB | 10.98 | 9.67 |  |
| LB | 0.46 | 0.39 |  |
| Data and definitions from Global Burden of Disease Database. 2021 | | | |
| *****Each value corresponds to mortality rates per 100.000 inhabitants (95% Uncertainty Interval). | | | |

|  | **SE Men** | **SE Women** | **Z-Score Men** | **Z-Score Women** |
| --- | --- | --- | --- | --- |
| **Bangladesh** | 1.76 | 0.98 | -0.01 | 0.02 |
| **Bhutan** | 1.83 | 0.98 | 0.00 | 0.00 |
| **India** | 2.35 | 1.37 | 0.49 | 0.43 |
| **Nepal** | 2.27 | 1.17 | 0.33 | 0.30 |
| **Pakistan** | 2.68 | 2.37 | 0.51 | 0.97 |

| **Supplemental Table 27. Age-standardised mortality rates (95% UI) for IHD attributable to Diet high in sugar-sweetened beverages, 2021.** | | | |
| --- | --- | --- | --- |
| **Country** | **Men*** | **Women*** | **Women-to-Men ratio** |
| **Bangladesh** | 0.02 | 0.01 | 0.55  -5.86  -0.06 |
| UB | 0.04 | 0.02 |  |
| LB | 0.00 | 0.00 |  |
| **Bhutan** | 0.03 | 0.02 | 0.57  -5.58  -0.05 |
| UB | 0.07 | 0.04 |  |
| LB | -0.01 | 0.00 |  |
| **India** | 0.13 | 0.08 | 0.57  -5.45  -0.06 |
| UB | 0.28 | 0.17 |  |
| LB | -0.03 | -0.02 |  |
| **Nepal** | 0.03 | 0.02 | 0.55  -6.52  -0.05 |
| UB | 0.08 | 0.05 |  |
| LB | -0.01 | 0.00 |  |
| **Pakistan** | 0.03 | 0.03 | 0.87  -9.97  -0.07 |
| UB | 0.08 | 0.06 |  |
| LB | -0.01 | -0.01 |  |
| Data and definitions from Global Burden of Disease Database. 2021 | | | |
| *****Each value corresponds to mortality rates per 100.000 inhabitants (95% Uncertainty Interval). Negative values in the UI indicate overlapping intervals for men and women. | | | |

|  | **SE Men** | **SE Women** | **Z-Score Men** | **Z-Score Women** |
| --- | --- | --- | --- | --- |
| **Bangladesh** | 0.01 | 0.01 | -0.43 | -0.46 |
| **Bhutan** | 0.02 | 0.01 | 0.00 | 0.00 |
| **India** | 0.08 | 0.05 | 1.24 | 1.22 |
| **Nepal** | 0.02 | 0.01 | 0.17 | 0.13 |
| **Pakistan** | 0.02 | 0.02 | 0.08 | 0.51 |

| **Supplemental Table 28. Age-standardised mortality rates (95% UI) for IHD attributable to Diet high in sodium, 2021.** | | | |
| --- | --- | --- | --- |
| **Country** | **Men*** | **Women*** | **Women-to-Men ratio** |
| **Bangladesh** | 7.41 | 4.27 | 0.58  155.16  0.00 |
| UB | 22.00 | 13.08 |  |
| LB | 0.08 | 0.03 |  |
| **Bhutan** | 8.25 | 4.11 | 0.50  125.64  0.00 |
| UB | 24.11 | 12.93 |  |
| LB | 0.10 | 0.02 |  |
| **India** | 11.60 | 4.33 | 0.37  18.69  0.00 |
| UB | 32.71 | 14.44 |  |
| LB | 0.77 | 0.07 |  |
| **Nepal** | 10.12 | 4.82 | 0.48  109.03  0.00 |
| UB | 28.98 | 15.09 |  |
| LB | 0.14 | 0.04 |  |
| **Pakistan** | 11.31 | 9.07 | 0.80  114.42  0.00 |
| UB | 33.34 | 26.62 |  |
| LB | 0.23 | 0.11 |  |
| Data and definitions from Global Burden of Disease Database. 2021 | | | |
| *****Each value corresponds to mortality rates per 100.000 inhabitants (95% Uncertainty Interval). | | | |

|  | **SE Men** | **SE Women** | **Z-Score Men** | **Z-Score Women** |
| --- | --- | --- | --- | --- |
| **Bangladesh** | 5.59 | 3.33 | -0.10 | 0.03 |
| **Bhutan** | 6.12 | 3.29 | 0.00 | 0.00 |
| **India** | 8.15 | 3.67 | 0.33 | 0.05 |
| **Nepal** | 7.36 | 3.84 | 0.19 | 0.14 |
| **Pakistan** | 8.45 | 6.76 | 0.29 | 0.66 |

| **Supplemental Table 29. Age-standardised mortality rates (95% UI) for IHD attributable to Diet high in red meat, 2021.** | | | |
| --- | --- | --- | --- |
| **Country** | **Men*** | **Women*** | **Women-to-Men ratio** |
| **Bangladesh** | -0.06 | -0.03 | 0.51  0.00  -40.91 |
| UB | 0.00 | 0.00 |  |
| LB | -0.15 | -0.07 |  |
| **Bhutan** | -0.12 | -0.06 | 0.53  -0.03  -18.54 |
| UB | 0.01 | 0.01 |  |
| LB | -0.28 | -0.15 |  |
| **India** | -0.08 | -0.04 | 0.56  -0.01  -8.21 |
| UB | 0.01 | 0.00 |  |
| LB | -0.18 | -0.10 |  |
| **Nepal** | 0.21 | 0.09 | 0.42  -8.20  -0.04 |
| UB | 0.88 | 0.40 |  |
| LB | -0.05 | -0.04 |  |
| **Pakistan** | 0.61 | 0.44 | 0.71  -166.14  -0.01 |
| UB | 1.62 | 1.20 |  |
| LB | -0.01 | -0.01 |  |
| Data and definitions from Global Burden of Disease Database. 2021 | | | |
| *****Each value corresponds to mortality rates per 100.000 inhabitants (95% Uncertainty Interval). Negative values in the UI indicate overlapping intervals for men and women. | | | |

|  | **SE Men** | **SE Women** | **Z-Score Men** | **Z-Score Women** |
| --- | --- | --- | --- | --- |
| **Bangladesh** | 0.04 | 0.02 | 0.61 | 0.64 |
| **Bhutan** | 0.07 | 0.04 | 0.00 | 0.00 |
| **India** | 0.05 | 0.03 | 0.44 | 0.38 |
| **Nepal** | 0.24 | 0.11 | 1.33 | 1.28 |
| **Pakistan** | 0.41 | 0.31 | 1.73 | 1.60 |

| **Supplemental Table 30. Age-standardised mortality rates (95% UI) for IHD attributable to Diet high in processed meat, 2021.** | | | |
| --- | --- | --- | --- |
| **Country** | **Men*** | **Women*** | **Women-to-Men ratio** |
| **Bangladesh** | 0.61 | 0.37 | 0.60  3.53  0.10 |
| UB | 1.14 | 0.70 |  |
| LB | 0.20 | 0.12 |  |
| **Bhutan** | 0.69 | 0.47 | 0.67  3.81  0.12 |
| UB | 1.32 | 0.88 |  |
| LB | 0.23 | 0.16 |  |
| **India** | 0.05 | 0.03 | 0.56  2.37  0.13 |
| UB | 0.07 | 0.04 |  |
| LB | 0.02 | 0.01 |  |
| **Nepal** | 0.88 | 0.56 | 0.64  3.57  0.12 |
| UB | 1.67 | 1.04 |  |
| LB | 0.29 | 0.19 |  |
| **Pakistan** | 0.99 | 0.97 | 0.97  4.91  0.18 |
| UB | 1.85 | 1.79 |  |
| LB | 0.37 | 0.33 |  |
| Data and definitions from Global Burden of Disease Database. 2021 | | | |
| *****Each value corresponds to mortality rates per 100.000 inhabitants (95% Uncertainty Interval). | | | |

|  | **SE Men** | **SE Women** | **Z-Score Men** | **Z-Score Women** |
| --- | --- | --- | --- | --- |
| **Bangladesh** | 0.24 | 0.15 | -0.22 | -0.41 |
| **Bhutan** | 0.28 | 0.18 | 0.00 | 0.00 |
| **India** | 0.01 | 0.01 | -2.33 | -2.39 |
| **Nepal** | 0.35 | 0.21 | 0.42 | 0.33 |
| **Pakistan** | 0.38 | 0.37 | 0.64 | 1.20 |

| **Supplemental Table 31: Population* in South Asia in 2005 and 2021, by sex, country. Data from World Bank.** | | | | |
| --- | --- | --- | --- | --- |
| **Country** | **2005** | | **2021** | |
|  | **Men** | **Women** | **Men** | **Women** |
| **Bangladesh** | 71,337,878 | 69,574,712 | 83,998,088 | 85,358,163 |
| **Bhutan** | 353,558 | 309,765 | 411,668 | 365,818 |
| **India** | 597,477,666 | 557,161,047 | 726,503,429 | 681,060,412 |
| **Nepal** | 13,098,713 | 13,186,397 | 14,370,965 | 15,664,025 |
| **Pakistan** | 89,942,008 | 84,430,090 | 116,815,852 | 114,586,264 |
| *Population is based on the de facto definition of population, which counts all residents regardless of legal status or citizenship. The values reported are midyear estimates. | | | | |
| World Bank staff estimates using the World Bank's total population and age/sex distributions of the United Nations Population Division's World Population Prospects: 2022 Revision. | | | | |
| Data and definition from World Bank (last accessed 28/10/2024). | | | | |

| **Supplemental Table 32: Women to men ratios of ASMR-to-ASPR index (95% UI) in 2005, 2010, 2015, 2021 in individuals of all ages, per 100,000 inhabitants. Stratified for sex and country. Data from GBD 2021.** | | | | |  |
| --- | --- | --- | --- | --- | --- |
| **Country** | **Women to men ratio** | | | |  |
|  |  |  |  |  |  |
|  | **2005** | **2010** | **2015** | **2021** |  |
|  |  |  |  |  |  |
| **Bangladesh** | 0.89 | 0.92 | 1.10 | 0.97 |  |
|  | (0.51 - 1.49) | (0.54 - 1.52) | (0.63 - 1.93) | (0.48 - 2.02) |  |
| **Bhutan** | 1.03 | 0.94 | 0.93 | 0.88 |  |
|  | (0.48 - 2.2) | (0.45 - 1.93) | (0.47 - 1.8) | (0.39 - 1.96) |  |
| **India** | 0.91 | 0.88 | 0.89 | 0.82 |  |
|  | (0.54 - 1.48) | (0.54 - 1.39) | (0.56 - 1.4) | (0.43 - 1.48) |  |
| **Nepal** | 1.17 | 1.04 | 0.95 | 0.88 |  |
|  | (0.66 - 2.14) | (0.59 - 1.86) | (0.48 - 1.84) | (0.42 - 1.91) |  |
| **Pakistan** | 1.51 | 1.40 | 1.46 | 1.43 |  |
|  | (0.83 - 2.91) | (0.71 - 2.82) | (0.7 - 3.04) | (0.6 - 3.46) |  |
| **South Asia**  **average** | 0.96 | 1.11 | 1.16 | 1.02 |  |
|  | (0.6 - 1.52) | (0.71 - 1.77) | (0.76 - 1.83) | (0.61 - 1.68) |  |
|  | | | | |  |
| ASMR= age standardized mortality rate  ASPR= age standardized prevalence rate  IHD= ischemic heart disease. GBD 2021 definition describes IHD as International Classification of Disease (ICD) classes I20-25.9, namely: angina pectoris, acute myocardial infarction, subsequent ST elevation (STEMI) and non-ST elevation (NSTEMI) myocardial infarction, certain current complications following ST elevation (STEMI) and non-ST elevation (NSTEMI) myocardial infarction (within the 28-day period), other acute ischemic heart disease, and chronic ischemic heart disease.  Data and definitions from Global Burden of Disease Database, 2021. | | | | |  |

| **Supplemental Table 33: GNI per Capita (USD) of South Asian countries, 2005 to 2021. GNI per Capita (USD) in 2005 and 2021, by country. Data from World Bank Databank.** | | | | | | | | | | | | | | | | | |
| --- | --- | --- | --- | --- | --- | --- | --- | --- | --- | --- | --- | --- | --- | --- | --- | --- | --- |
| **Country** | **2005** | **2006** | **2007** | **2008** | **2009** | **2010** | **2011** | **2012** | **2013** | **2014** | **2015** | **2016** | **2017** | **2018** | **2019** | **2020** | **2021** |
| **Bangladesh** | 540 | 550 | 580 | 640 | 700 | 780 | 870 | 950 | 1010 | 1080 | 1200 | 1400 | 1640 | 2020 | 2220 | 2320 | 2570 |
| **Bhutan** | 1260 | 1390 | 1700 | 1850 | 1980 | 2180 | 2430 | 2620 | 2630 | 2670 | 2740 | 2880 | 2960 | 3150 | 3370 | 2040 | 3290 |
| **India** | 700 | 780 | 910 | 990 | 1110 | 1210 | 1350 | 1460 | 1500 | 1540 | 1580 | 1670 | 1790 | 1970 | 2070 | 1900 | 2180 |
| **Nepal** | 300 | 330 | 370 | 430 | 480 | 530 | 620 | 750 | 840 | 860 | 870 | 860 | 970 | 1120 | 1240 | 1190 | 1230 |
| **Pakistan** | 840 | 890 | 940 | 1000 | 1020 | 1000 | 1020 | 1110 | 1210 | 1250 | 1280 | 1360 | 1450 | 1560 | 1520 | 1370 | 1470 |
| *GNI is based on the de facto definition of population, which counts all residents regardless of legal status or citizenship. The values reported are midyear estimates. | | | | | | | | | | | | | | | | | |
| Classifications from 2023-24 World Bank data. South Asian countries classified as low and middle-income (LMICs) with gross domestic product (GNI) per capita of $4,515 or less. | | | | | | | | | | | | | | | | | |
| Data and definition from World bank Databank, Atlas methos (current US$). | | | | | | | | | | | | | | | | | |

| **Supplemental Table 34: Age-standardised prevalence rate of IHD per 100,000 inhabitants (95% UI)** | | | | | | | | | | | | | | | | |
| --- | --- | --- | --- | --- | --- | --- | --- | --- | --- | --- | --- | --- | --- | --- | --- | --- |
| **Country** | **2005** | | **2006** | | **2007** | | **2008** | | **2009** | | **2010** | | **2011** | | **2012** | |
|  | **Men** | **Women** | **Men** | **Women** | **Men** | **Women** | **Men** | **Women** | **Men** | **Women** | **Men** | **Women** | **Men** | **Women** | **Men** | **Women** |
| **Bangladesh** | 4685.91 | 2771.06 | 4692.74 | 2772.68 | 4699.15 | 2775.82 | 4706.28 | 2780.71 | 4715.28 | 2787.60 | 4727.30 | 2796.73 | 4752.67 | 2814.61 | 4793.59 | 2843.08 |
|  | (4318.87 - 5070.59) | (2552.05 - 3006.87) | (4332.13 - 5073.61) | (2559.37 - 3007.32) | (4341.11 - 5072.72) | (2564.69 - 3005.65) | (4362.9 - 5086.84) | (2566.23 - 3015.92) | (4369.66 - 5103.35) | (2565.45 - 3029.17) | (4374 - 5125.59) | (2562.54 - 3038.76) | (4388.56 - 5136.34) | (2589.89 - 3058.88) | (4440.96 - 5175.99) | (2613.93 - 3095.37) |
| **Bhutan** | 5188.94 | 3224.97 | 5200.81 | 3229.03 | 5213.73 | 3233.17 | 5226.74 | 3237.26 | 5238.91 | 3241.17 | 5249.28 | 3244.76 | 5258.76 | 3248.81 | 5268.50 | 3253.97 |
|  | (4790.64 - 5653.21) | (2977.46 - 3527.30) | (4810.25 - 5647.87) | (2989.22 - 3532.24) | (4828.4 - 5662.4) | (2991.6 - 3539.63) | (4848.33 - 5687.4) | (2994.77 - 3541.54) | (4847.1 - 5689.92) | (2988.45 - 3545.82) | (4843.96 - 5707.96) | (2972.95 - 3556.22) | (4859.06 - 5697.15) | (2992.69 - 3553.28) | (4870.26 - 5700.47) | (3009.07 - 3547.78) |
| **India** | 5189.58 | 3319.41 | 5213.79 | 3325.68 | 5240.23 | 3334.18 | 5266.15 | 3344.19 | 5288.23 | 3354.90 | 5303.69 | 3365.54 | 5317.25 | 3378.13 | 5333.20 | 3394.46 |
|  | (4538.87 - 5927.17) | (2881.04 - 3798.31) | (4561.51 - 5952.79) | (2886.78 - 3808.67) | (4586.02 - 5980.24) | (2894.73 - 3820.66) | (4609.74 - 6006.58) | (2904.07 - 3830.57) | (4629.62 - 6028.36) | (2913.89 - 3841.47) | (4643.3 - 6042.89) | (2924.94 - 3853.17) | (4654.41 - 6055.62) | (2938 - 3866.35) | (4667.9 - 6068.6) | (2955.11 - 3883.71) |
| **Nepal** | 4766.57 | 2929.20 | 4764.59 | 2923.58 | 4752.50 | 2919.74 | 4735.78 | 2916.35 | 4719.89 | 2912.07 | 4710.29 | 2905.56 | 4705.43 | 2895.09 | 4700.30 | 2881.54 |
|  | (4372.47 - 5164.79) | (2700.79 - 3183.39) | (4393.25 - 5147.68) | (2697.87 - 3180.51) | (4396.48 - 5124.67) | (2692.86 - 3176.03) | (4389.84 - 5121.55) | (2690.86 - 3181.7) | (4369.38 - 5107.61) | (2690.35 - 3191.27) | (4336.05 - 5104.68) | (2678.3 - 3182.76) | (4346.57 - 5087.43) | (2678.12 - 3159.98) | (4359.59 - 5074.13) | (2666.12 - 3136.37) |
| **Pakistan** | 6225.66 | 3824.69 | 6233.40 | 3839.30 | 6239.59 | 3862.98 | 6244.68 | 3888.61 | 6249.04 | 3908.87 | 6253.01 | 3916.68 | 6257.32 | 3912.51 | 6262.13 | 3903.52 |
|  | (5473.62 - 7069.97) | (3327.40 - 4381.71) | (5463.24 - 7076.4) | (3342.84 - 4395.37) | (5466.86 - 7073.38) | (3364.08 - 4415.62) | (5457.58 - 7071.08) | (3388.82 - 4438) | (5453.38 - 7076.06) | (3415.52 - 4451.45) | (5461.78 - 7078.21) | (3417.68 - 4449.65) | (5464.9 - 7083.67) | (3416.95 - 4443.27) | (5475.59 - 7091.92) | (3409.85 - 4430.42) |
|  |  |  |  |  |  |  |  |  |  |  |  |  |  |  |  |  |
| **Overall** | 26056.66 | 16069.33 | 26105.33 | 16090.26 | 26145.20 | 16125.88 | 26179.62 | 16167.12 | 26211.35 | 16204.60 | 26243.58 | 16229.28 | 26291.43 | 16249.15 | 26357.71 | 16276.56 |
|  | (23494.47 - 28885.73) | (14438.74 - 17897.58) | (23560.39 - 28898.35) | (14476.08 - 17924.12) | (23618.87 - 28913.42) | (14507.95 - 17957.59) | (23668.39 - 28973.44) | (14544.76 - 18007.73) | (23669.14 - 29005.31) | (14573.66 - 18059.17) | (23659.09 - 29059.32) | (14556.41 - 18080.56) | (23713.5 - 29060.2) | (14615.64 - 18081.76) | (23814.3 - 29111.11) | (14654.07 - 18093.65) |
| **South Asia average** | 5236.14 | 3315.30 | 5221.07 | 3218.05 | 5229.04 | 3225.18 | 5235.92 | 3233.42 | 5242.27 | 3240.92 | 5248.72 | 3245.86 | 5258.29 | 3249.83 | 5271.54 | 3255.31 |
|  | (4617.87 - 5933.63) | (2894.35 - 3777.37) | (4712.08 - 5779.67) | (2895.22 - 3584.82) | (4723.77 - 5782.68) | (2901.59 - 3591.52) | (4733.68 - 5794.69) | (2908.95 - 3601.55) | (4733.83 - 5801.06) | (2914.73 - 3611.83) | (4731.82 - 5811.86) | (2911.28 - 3616.11) | (4742.7 - 5812.04) | (2923.13 - 3616.35) | (4762.86 - 5822.22) | (2930.81 - 3618.73) |

| **Supplemental Table 35: Age-standardised prevalence rate of IHD per 100,000 inhabitants (95% UI), continued** | | | | | | | | | | | | | | | | | | |
| --- | --- | --- | --- | --- | --- | --- | --- | --- | --- | --- | --- | --- | --- | --- | --- | --- | --- | --- |
| **Country** | **2013** | | **2014** | | **2015** | | **2016** | | **2017** | | **2018** | | **2019** | | **2020** | | **2021** | |
|  | **Men** | **Women** | **Men** | **Women** | **Men** | **Women** | **Men** | **Women** | **Men** | **Women** | **Men** | **Women** | **Men** | **Women** | **Men** | **Women** | **Men** | **Women** |
| **Bangladesh** | 4839.03 | 2875.47 | 4877.97 | 2905.07 | 4899.38 | 2925.19 | 4909.90 | 2941.10 | 4921.54 | 2959.26 | 4931.31 | 2974.75 | 4936.26 | 2982.67 | 4929.94 | 2980.66 | 4876.19 | 3009.09 |
|  | (4482.2 - 5217.92) | (2644.87 - 3135.54) | (4520.73 - 5261.89) | (2665.92 - 3176.2) | (4533.83 - 5303.27) | (2672.74 - 3202.7) | (4569.21 - 5306.22) | (2695.29 - 3217.44) | (4576.75 - 5317.12) | (2722.36 - 3231.65) | (4563.05 - 5341.44) | (2731.94 - 3242.01) | (4550.66 - 5360.23) | (2740.58 - 3245.86) | (4528.39 - 5359.01) | (2716.42 - 3261.51) | (4410.08 - 5407.42) | (2682.72 - 3355.03) |
| **Bhutan** | 5277.99 | 3259.86 | 5286.68 | 3266.13 | 5294.06 | 3272.42 | 5301.43 | 3282.65 | 5309.49 | 3297.44 | 5316.81 | 3311.43 | 5321.93 | 3319.31 | 5327.44 | 3314.27 | 5340.86 | 3366.69 |
|  | (4885.29 - 5715.92) | (3016.31 - 3548.32) | (4885.18 - 5715.81) | (3019.25 - 3552.09) | (4888.92 - 5752.57) | (3018.97 - 3559.89) | (4912.87 - 5766.1) | (3026.97 - 3569.21) | (4920 - 5778.06) | (3035.17 - 3599.07) | (4897.41 - 5793.7) | (3032.31 - 3626.41) | (4883.63 - 5819.62) | (3017.48 - 3649.64) | (4865.23 - 5820.74) | (3015.64 - 3658.81) | (4768.35 - 5963.44) | (2999.86 - 3791.54) |
| **India** | 5348.20 | 3413.08 | 5359.00 | 3432.10 | 5361.34 | 3448.87 | 5352.75 | 3472.40 | 5338.07 | 3506.01 | 5323.37 | 3537.61 | 5315.33 | 3554.99 | 5317.39 | 3555.22 | 5322.73 | 3659.84 |
|  | (4679.8 - 6085.3) | (2974.08 - 3905.11) | (4687.75 - 6093.64) | (2989.55 - 3927.72) | (4683.82 - 6103.39) | (3000.13 - 3948.38) | (4660.94 - 6113.06) | (3014.05 - 3979.69) | (4633.32 - 6113) | (3026.65 - 4032.73) | (4605.28 - 6122.76) | (3038.25 - 4088.29) | (4581.07 - 6124.85) | (3045.25 - 4137.43) | (4594.78 - 6132.38) | (3048.44 - 4150.1) | (4457.39 - 6410.86) | (3094.23 - 4460.36) |
| **Nepal** | 4695.23 | 2867.51 | 4690.55 | 2855.62 | 4686.60 | 2848.49 | 4681.69 | 2845.45 | 4675.46 | 2843.93 | 4670.17 | 2843.68 | 4668.09 | 2844.46 | 4676.59 | 2849.45 | 4696.58 | 2888.46 |
|  | (4348.82 - 5071.02) | (2653.77 - 3118.78) | (4349.51 - 5066.53) | (2638.66 - 3105.27) | (4349.05 - 5073.56) | (2622.67 - 3096.07) | (4335.62 - 5056.36) | (2619.76 - 3093.66) | (4319.9 - 5066.28) | (2605.15 - 3097.56) | (4300.45 - 5080.57) | (2604.01 - 3104.89) | (4263.86 - 5109.64) | (2589.6 - 3112.34) | (4307.8 - 5084.79) | (2603.56 - 3104.63) | (4257.71 - 5196.62) | (2602.81 - 3235.96) |
| **Pakistan** | 6266.78 | 3892.90 | 6270.60 | 3883.56 | 6272.75 | 3878.66 | 6274.02 | 3880.87 | 6275.52 | 3887.96 | 6276.99 | 3896.00 | 6278.15 | 3900.84 | 6282.35 | 3900.88 | 6274.03 | 3924.14 |
|  | (5483.17 - 7102.1) | (3402.63 - 4418.4) | (5502.72 - 7111.51) | (3394.77 - 4408.97) | (5512.19 - 7120.29) | (3391.25 - 4411.48) | (5489.04 - 7127.93) | (3384.89 - 4418.26) | (5463.2 - 7149.95) | (3381.55 - 4453.38) | (5424.3 - 7180.44) | (3355.62 - 4505.61) | (5398.81 - 7229.55) | (3345.38 - 4535.66) | (5414.88 - 7221.68) | (3341.59 - 4520.23) | (5292.03 - 7406.30) | (3307.36 - 4733.61) |
|  |  |  |  |  |  |  |  |  |  |  |  |  |  |  |  |  |  |  |
| **Overall** | 26427.23 | 16308.82 | 26484.80 | 16342.49 | 26514.13 | 16373.63 | 26519.80 | 16422.48 | 26520.08 | 16494.58 | 26518.65 | 16563.47 | 26519.77 | 16602.27 | 26533.71 | 16600.48 | 26510.39 | 16848.22 |
|  | (23879.28 - 29192.26) | (14691.66 - 18126.14) | (23945.89 - 29249.38) | (14708.15 - 18170.25) | (23967.8 - 29353.08) | (14705.77 - 18218.52) | (23967.68 - 29369.67) | (14740.95 - 18278.26) | (23913.17 - 29424.4) | (14770.89 - 18414.39) | (23790.49 - 29518.9) | (14762.14 - 18567.21) | (23678.04 - 29643.89) | (14738.29 - 18680.93) | (23711.07 - 29618.59) | (14725.66 - 18695.27) | (23185.56 - 30384.64) | (14686.98 - 19576.50) |
| **South Asia average** | 5285.45 | 3261.76 | 5296.96 | 3268.50 | 5302.83 | 3274.73 | 5303.96 | 3284.50 | 5304.02 | 3298.92 | 5303.73 | 3312.69 | 5303.95 | 3320.45 | 5306.74 | 3320.10 | 5347.44 | 3609.07 |
|  | (4775.86 - 5838.45) | (2938.33 - 3625.23) | (4789.18 - 5849.88) | (2941.63 - 3634.05) | (4793.56 - 5870.62) | (2941.15 - 3643.7) | (4793.54 - 5873.93) | (2948.19 - 3655.65) | (4782.63 - 5884.88) | (2954.18 - 3682.88) | (4758.1 - 5903.78) | (2952.43 - 3713.44) | (4735.61 - 5928.78) | (2947.66 - 3736.19) | (4742.21 - 5923.72) | (2945.13 - 3739.05) | (4540.29 - 6343.90) | (3083.13 - 4359.80) |

| **Supplemental Table 36: Age-standardised mortality rate of IHD per 100,000 inhabitants (95% UI)** | | | | | | | | | | | | | | | | |
| --- | --- | --- | --- | --- | --- | --- | --- | --- | --- | --- | --- | --- | --- | --- | --- | --- |
|  |  |  |  |  |  |  |  |  |  |  |  |  |  |  |  |  |
| **Country** | **2005** | | **2006** | | **2007** | | **2008** | | **2009** | | **2010** | | **2011** | | **2012** | |
|  | **Men** | **Women** | **Men** | **Women** | **Men** | **Women** | **Men** | **Women** | **Men** | **Women** | **Men** | **Women** | **Men** | **Women** | **Men** | **Women** |
| **Bangladesh** | 145.17 | 76.10 | 150.88 | 76.80 | 152.05 | 77.24 | 147.70 | 76.79 | 145.40 | 77.43 | 145.85 | 79.69 | 131.49 | 77.64 | 121.60 | 79.07 |
|  | (126.54 - 168.35) | (59.88 - 94.99) | (131.41 - 174.51) | (60.94 - 95.81) | (133.66 - 176.28) | (61.46 - 95.1) | (129.65 - 170.61) | (60.7 - 94.11) | (127.48 - 168.42) | (61.23 - 94.27) | (127.26 - 167.42) | (63.1 - 96.96) | (113.8 - 149.71) | (63.63 - 93.16) | (103.09 - 140.74) | (65.62 - 93.8) |
| **Bhutan** | 132.07 | 84.26 | 132.93 | 83.05 | 134.23 | 82.08 | 134.91 | 81.17 | 136.73 | 80.28 | 137.48 | 80.09 | 137.38 | 78.83 | 139.75 | 81.11 |
|  | (95.80 - 170.30) | (60.60 - 111.11) | (97.12 - 170.09) | (59.42 - 109.12) | (99.03 - 172.3) | (58.61 - 108.34) | (100.12 - 173.03) | (58.27 - 108.13) | (101.77 - 173.87) | (57.97 - 106.49) | (105.04 - 172.92) | (57.69 - 105.76) | (103.67 - 171.41) | (57.19 - 105.46) | (108.62 - 173.43) | (59.81 - 108.25) |
| **India** | 168.68 | 97.84 | 171.82 | 102.54 | 179.09 | 106.30 | 182.33 | 106.94 | 180.81 | 104.11 | 185.62 | 103.31 | 190.44 | 106.34 | 192.30 | 113.14 |
|  | (152.90 - 184.64) | (84.07 - 110.02) | (155.46 - 188.05) | (88.12 - 114.34) | (163.07 - 194.49) | (92.05 - 117.28) | (166 - 199.7) | (92.86 - 118.64) | (165.64 - 196) | (91.57 - 115.6) | (170.24 - 202.02) | (90.57 - 114.58) | (173.9 - 205.37) | (93.8 - 117.59) | (177.01 - 207.86) | (101.2 - 125.73) |
| **Nepal** | 140.72 | 100.78 | 144.30 | 100.95 | 148.71 | 102.08 | 152.79 | 102.63 | 157.51 | 102.95 | 161.43 | 103.78 | 167.82 | 103.88 | 174.60 | 107.63 |
|  | (110.54 - 169.02) | (80.99 - 123.62) | (115.05 - 171.92) | (80.83 - 123.55) | (118.32 - 177.48) | (82.58 - 124.34) | (122.82 - 180.22) | (82.1 - 125.77) | (127.23 - 186.19) | (82.57 - 126.07) | (131.85 - 190.93) | (82.7 - 128.47) | (136.65 - 201.22) | (81.61 - 129.28) | (141.95 - 213.54) | (83.89 - 135.31) |
| **Pakistan** | 178.04 | 164.96 | 179.18 | 162.85 | 180.11 | 161.48 | 182.21 | 161.77 | 184.44 | 162.59 | 186.53 | 163.60 | 189.30 | 165.82 | 193.64 | 171.88 |
|  | (142.62 - 210.96) | (140.00 - 195.59) | (146.2 - 214.65) | (135.88 - 192.48) | (145.48 - 220.63) | (133.44 - 192.77) | (142.38 - 222.77) | (133.28 - 192.4) | (147.21 - 227.13) | (132.84 - 197.04) | (145.33 - 229.21) | (132.88 - 197.95) | (146.8 - 239.61) | (135 - 202.08) | (149.72 - 240.46) | (137.03 - 210.25) |
|  |  |  |  |  |  |  |  |  |  |  |  |  |  |  |  |  |
| **Overall** | 764.68 | 523.94 | 779.12 | 526.20 | 794.19 | 529.19 | 799.93 | 529.29 | 804.88 | 527.36 | 816.90 | 530.47 | 816.43 | 532.52 | 821.88 | 552.83 |
|  | (628.40 - 903.27) | (425.54 - 635.33) | (645.24 - 919.22) | (425.19 - 635.3) | (659.56 - 941.18) | (428.15 - 637.83) | (660.96 - 946.33) | (427.2 - 639.05) | (669.33 - 951.61) | (426.17 - 639.47) | (679.72 - 962.5) | (426.94 - 643.71) | (674.8 - 967.32) | (431.23 - 647.57) | (680.39 - 976.03) | (447.53 - 673.34) |
| **South Asia average** | 167.12 | 101.63 | 1407.36 | 975.60 | 1436.33 | 981.13 | 1452.16 | 981.79 | 1464.36 | 977.29 | 1487.95 | 981.25 | 1501.37 | 987.39 | 1522.16 | 1026.58 |
|  | (153.08 - 182.18) | (88.74 - 113.45) | (129.05 - 183.84) | (85.04 - 127.06) | (131.91 - 188.24) | (85.63 - 127.57) | (132.19 - 189.27) | (85.44 - 127.81) | (133.87 - 190.32) | (85.23 - 127.89) | (135.94 - 192.5) | (85.39 - 128.74) | (134.96 - 193.46) | (86.25 - 129.51) | (136.08 - 195.21) | (89.51 - 134.67) |

| **Supplemental Table 37: Age-standardised mortality rate of IHD per 100,000 inhabitants (95% UI), continued** | | | | | | | | | | | | | | | | | | | |  |  |
| --- | --- | --- | --- | --- | --- | --- | --- | --- | --- | --- | --- | --- | --- | --- | --- | --- | --- | --- | --- | --- | --- |
|  |  |  |  |  |  |  |  |  |  |  |  |  |  |  |  |  |  |  |  |  | |
| **Country** | **2013** | | **2014** | | **2015** | | **2016** | | **2017** | | **2018** | | **2019** | | **2020** | | **2021** | | |  | |
|  | **Men** | **Women** | **Men** | **Women** | **Men** | **Women** | **Men** | **Women** | **Men** | **Women** | **Men** | **Women** | **Men** | **Women** | **Men** | **Women** | **Men** | **Women** |  | |  |
| **Bangladesh** | 121.22 | 79.04 | 128.07 | 83.02 | 125.50 | 82.65 | 126.43 | 82.49 | 133.83 | 82.65 | 134.16 | 82.49 | 134.00 | 81.72 | 133.65 | 81.07 | 133.61 | 80.41 |  | |  |
|  | (102.43 - 139.95) | (64.01 - 94.22) | (107.86 - 150.75) | (67.42 - 99.82) | (104.27 - 148.72) | (65.96 - 101.16) | (104.37 - 152.09) | (64.33 - 101.18) | (108.15 - 160.97) | (64.18 - 103) | (107.92 - 163.41) | (63.82 - 103.77) | (105.27 - 164.56) | (62.67 - 103.43) | (104.27 - 164.86) | (61.35 - 103.14) | (104.20 - 165.80) | (60.94 - 104.61) |  | |  |
| **Bhutan** | 139.55 | 80.13 | 141.75 | 82.46 | 141.09 | 80.77 | 141.54 | 80.54 | 141.29 | 79.16 | 142.06 | 79.41 | 142.56 | 79.36 | 143.00 | 79.48 | 143.04 | 79.08 |  | |  |
|  | (108.85 - 173.61) | (59.39 - 106.64) | (110.83 - 175.99) | (61.92 - 107.19) | (110.73 - 174.8) | (59.61 - 104.34) | (111.5 - 175.89) | (59 - 105.41) | (109.02 - 176.4) | (57.14 - 104.22) | (109.56 - 177.88) | (57.51 - 104.5) | (109.33 - 178.25) | (57.38 - 104.99) | (109.04 - 179.18) | (56.67 - 106.86) | (109.00 - 178.93) | (55.37 - 107.54) |  | |  |
| **India** | 203.31 | 118.71 | 217.45 | 123.20 | 207.10 | 118.43 | 201.14 | 119.82 | 194.64 | 116.10 | 196.19 | 116.21 | 197.31 | 115.25 | 197.53 | 112.69 | 197.53 | 110.77 |  | |  |
|  | (187.47 - 219.94) | (105.61 - 131.21) | (201.15 - 234.55) | (110.33 - 137.09) | (190.27 - 224.82) | (106.83 - 130.53) | (184.81 - 218.23) | (107.83 - 131.5) | (179.64 - 210.09) | (104.28 - 126.04) | (180.34 - 213.02) | (105.03 - 127.02) | (179.62 - 215.85) | (103.33 - 129.46) | (177.87 - 218.82) | (99.98 - 126.56) | (175.08 - 223.59) | (96.09 - 124.77) |  | |  |
| **Nepal** | 176.86 | 105.96 | 180.00 | 107.97 | 180.15 | 104.08 | 180.80 | 103.28 | 177.82 | 97.61 | 178.66 | 98.06 | 178.93 | 97.87 | 179.97 | 98.18 | 180.62 | 98.18 |  | |  |
|  | (143.06 - 218.88) | (81.26 - 134.99) | (143.69 - 224.14) | (79.71 - 140.34) | (144.56 - 224.2) | (76.95 - 137.43) | (144.08 - 225.82) | (74.7 - 137.53) | (141.58 - 218.6) | (71.32 - 130.84) | (141.52 - 220.09) | (71.98 - 133) | (141.49 - 220.49) | (72.26 - 132.76) | (140.95 - 222.72) | (72.21 - 134.61) | (140.84 - 224.56) | (72.14 - 134.93) |  | |  |
| **Pakistan** | 196.88 | 176.55 | 199.19 | 180.69 | 198.33 | 178.55 | 199.44 | 180.47 | 196.33 | 175.55 | 194.88 | 174.82 | 194.52 | 174.22 | 194.08 | 173.92 | 193.50 | 172.65 |  | |  |
|  | (155.99 - 243.55) | (141.37 - 214.04) | (155.72 - 251.02) | (146.33 - 220.72) | (153.12 - 251.38) | (141.16 - 221.44) | (152.92 - 253.23) | (142.28 - 226.39) | (150.32 - 258.09) | (139.62 - 227.98) | (149.39 - 248.16) | (135.24 - 219.29) | (150.04 - 252.82) | (136.54 - 223.12) | (145.81 - 257.68) | (134.11 - 229.71) | (146.39 - 247.52) | (133.43 - 225.97) |  | |  |
|  |  |  |  |  |  |  |  |  |  |  |  |  |  |  |  |  |  |  |  | |  |
| **Overall** | 837.82 | 560.38 | 866.46 | 577.34 | 852.18 | 564.49 | 849.36 | 566.61 | 843.92 | 551.07 | 845.96 | 551.00 | 847.31 | 548.42 | 848.24 | 545.35 | 848.30 | 541.09 |  | |  |
|  | (697.8 - 995.93) | (451.63 - 681.11) | (719.24 - 1036.45) | (465.72 - 705.15) | (702.95 - 1023.91) | (450.51 - 694.89) | (697.68 - 1025.25) | (448.15 - 702.01) | (688.71 - 1024.16) | (436.54 - 692.08) | (688.73 - 1022.56) | (433.58 - 687.58) | (685.75 - 1031.98) | (432.17 - 693.75) | (677.94 - 1043.27) | (424.33 - 700.88) | (675.51 - 1040.40) | (417.97 - 697.82) |  | |  |
| **South Asia average** | 1554.42 | 1041.73 | 1604.85 | 1071.66 | 1578.86 | 1046.34 | 1572.28 | 1050.72 | 1554.01 | 1019.48 | 1557.75 | 1019.51 | 1560.63 | 1015.12 | 1562.83 | 1009.62 | 190.07 | 112.14 |  | |  |
|  | (139.56 - 199.19) | (90.33 - 136.22) | (143.85 - 207.29) | (93.14 - 141.03) | (140.59 - 204.78) | (90.1 - 138.98) | (139.54 - 205.05) | (89.63 - 140.4) | (137.74 - 204.83) | (87.31 - 138.42) | (137.75 - 204.51) | (86.72 - 137.52) | (137.15 - 206.4) | (86.43 - 138.75) | (135.59 - 208.65) | (84.87 - 140.18) | (169.87 - 212.11) | (99.08 - 124.35) |  | |  |

| **Supplemental Table 38: ASMR-to-ASPR index % (95% UI)** | | | | | | | | | | | | | | | | |  |
| --- | --- | --- | --- | --- | --- | --- | --- | --- | --- | --- | --- | --- | --- | --- | --- | --- | --- |
|  |  |  |  |  |  |  |  |  |  |  |  |  |  |  |  |  |  |
| **Country** | **2005** | | **2006** | | **2007** | | **2008** | | **2009** | | **2010** | | **2011** | | **2012** | |  |
|  | **Men** | **Women** | **Men** | **Women** | **Men** | **Women** | **Men** | **Women** | **Men** | **Women** | **Men** | **Women** | **Men** | **Women** | **Men** | **Women** |  |
| **Bangladesh** | 3.10 | 2.75 | 3.22 | 2.77 | 3.24 | 2.78 | 3.14 | 2.76 | 3.08 | 2.78 | 3.09 | 2.85 | 2.77 | 2.76 | 2.54 | 2.78 |  |
|  | (2.50 - 3.90) | (1.99 - 3.72) | (3.03 - 3.44) | (2.38 - 3.19) | (3.08 - 3.47) | (2.4 - 3.16) | (2.97 - 3.35) | (2.37 - 3.12) | (2.92 - 3.3) | (2.39 - 3.11) | (2.91 - 3.27) | (2.46 - 3.19) | (2.59 - 2.91) | (2.46 - 3.05) | (2.32 - 2.72) | (2.51 - 3.03) |  |
| **Bhutan** | 2.55 | 2.61 | 2.56 | 2.57 | 2.57 | 2.54 | 2.58 | 2.51 | 2.61 | 2.48 | 2.62 | 2.47 | 2.61 | 2.43 | 2.65 | 2.49 |  |
|  | (1.69 - 3.55) | (1.72 - 3.73) | (2.02 - 3.01) | (1.99 - 3.09) | (2.05 - 3.04) | (1.96 - 3.06) | (2.06 - 3.04) | (1.95 - 3.05) | (2.1 - 3.06) | (1.94 - 3) | (2.17 - 3.03) | (1.94 - 2.97) | (2.13 - 3.01) | (1.91 - 2.97) | (2.23 - 3.04) | (1.99 - 3.05) |  |
| **India** | 3.25 | 2.95 | 3.30 | 3.08 | 3.42 | 3.19 | 3.46 | 3.20 | 3.42 | 3.10 | 3.50 | 3.07 | 3.58 | 3.15 | 3.61 | 3.33 |  |
|  | (2.58 - 4.07) | (2.21 - 3.82) | (3.41 - 3.16) | (3.05 - 3) | (3.56 - 3.25) | (3.18 - 3.07) | (3.6 - 3.32) | (3.2 - 3.1) | (3.58 - 3.25) | (3.14 - 3.01) | (3.67 - 3.34) | (3.1 - 2.97) | (3.74 - 3.39) | (3.19 - 3.04) | (3.79 - 3.43) | (3.42 - 3.24) |  |
| **Nepal** | 2.95 | 3.44 | 3.03 | 3.45 | 3.13 | 3.50 | 3.23 | 3.52 | 3.34 | 3.54 | 3.43 | 3.57 | 3.57 | 3.59 | 3.71 | 3.74 |  |
|  | (2.14 - 3.87) | (2.54 - 4.58) | (2.62 - 3.34) | (3 - 3.88) | (2.69 - 3.46) | (3.07 - 3.91) | (2.8 - 3.52) | (3.05 - 3.95) | (2.91 - 3.65) | (3.07 - 3.95) | (3.04 - 3.74) | (3.09 - 4.04) | (3.14 - 3.96) | (3.05 - 4.09) | (3.26 - 4.21) | (3.15 - 4.31) |  |
| **Pakistan** | 2.86 | 4.31 | 2.87 | 4.24 | 2.89 | 4.18 | 2.92 | 4.16 | 2.95 | 4.16 | 2.98 | 4.18 | 3.03 | 4.24 | 3.09 | 4.40 |  |
|  | (2.02 - 3.85) | (3.20 - 5.88) | (2.68 - 3.03) | (4.06 - 4.38) | (2.66 - 3.12) | (3.97 - 4.37) | (2.61 - 3.15) | (3.93 - 4.34) | (2.7 - 3.21) | (3.89 - 4.43) | (2.66 - 3.24) | (3.89 - 4.45) | (2.69 - 3.38) | (3.95 - 4.55) | (2.73 - 3.39) | (4.02 - 4.75) |  |
|  |  |  |  |  |  |  |  |  |  |  |  |  |  |  |  |  |  |
| **Overall** | 2.93 | 3.26 | 2.98 | 3.27 | 3.04 | 3.28 | 3.06 | 3.27 | 3.07 | 3.25 | 3.11 | 3.27 | 3.11 | 3.28 | 3.12 | 3.40 |  |
|  | (2.18 - 3.84) | (2.38 - 4.40) | (2.74 - 3.18) | (2.94 - 3.54) | (2.79 - 3.26) | (2.95 - 3.55) | (2.79 - 3.27) | (2.94 - 3.55) | (2.83 - 3.28) | (2.92 - 3.54) | (2.87 - 3.31) | (2.93 - 3.56) | (2.85 - 3.33) | (2.95 - 3.58) | (2.86 - 3.35) | (3.05 - 3.72) |  |
| **South Asia average** | 3.19 | 3.07 | 2.99 | 3.22 | 3.05 | 3.24 | 3.07 | 3.23 | 3.08 | 3.21 | 3.12 | 3.23 | 3.11 | 3.23 | 3.12 | 3.35 |  |
|  | (2.58 - 3.95) | (2.35 - 3.92) | (2.75 - 3.2) | (2.9 - 3.51) | (2.81 - 3.27) | (2.91 - 3.51) | (2.81 - 3.28) | (2.9 - 3.51) | (2.84 - 3.29) | (2.89 - 3.5) | (2.89 - 3.32) | (2.9 - 3.52) | (2.86 - 3.33) | (2.91 - 3.54) | (2.87 - 3.36) | (3.02 - 3.68) |  |

| **Supplemental Table 39: ASMR-to-ASPR index % (95% UI), continued** | | | | | | | | | | | | | | | | | | |  |
| --- | --- | --- | --- | --- | --- | --- | --- | --- | --- | --- | --- | --- | --- | --- | --- | --- | --- | --- | --- |
|  |  |  |  |  |  |  |  |  |  |  |  |  |  |  |  |  |  |  |  |
| **Country** | **2013** | | **2014** | | **2015** | | **2016** | | **2017** | | **2018** | | **2019** | | **2020** | | **2021** | |  |
|  | **Men** | **Women** | **Men** | **Women** | **Men** | **Women** | **Men** | **Women** | **Men** | **Women** | **Men** | **Women** | **Men** | **Women** | **Men** | **Women** | **Men** | **Women** |  |
| **Bangladesh** | 2.51 | 2.75 | 2.63 | 2.86 | 2.56 | 2.83 | 2.57 | 2.80 | 2.72 | 2.79 | 2.72 | 2.77 | 2.71 | 2.74 | 2.71 | 2.72 | 2.74 | 2.67 |  |
|  | (2.29 - 2.68) | (2.42 - 3.01) | (2.39 - 2.86) | (2.53 - 3.14) | (2.3 - 2.8) | (2.47 - 3.16) | (2.28 - 2.87) | (2.39 - 3.14) | (2.36 - 3.03) | (2.36 - 3.19) | (2.37 - 3.06) | (2.34 - 3.2) | (2.31 - 3.07) | (2.29 - 3.19) | (2.3 - 3.08) | (2.26 - 3.16) | (1.93 - 3.76) | (1.82 - 3.90) |  |
| **Bhutan** | 2.64 | 2.46 | 2.68 | 2.52 | 2.67 | 2.47 | 2.67 | 2.45 | 2.66 | 2.40 | 2.67 | 2.40 | 2.68 | 2.39 | 2.68 | 2.40 | 2.68 | 2.35 |  |
|  | (2.23 - 3.04) | (1.97 - 3.01) | (2.27 - 3.08) | (2.05 - 3.02) | (2.26 - 3.04) | (1.97 - 2.93) | (2.27 - 3.05) | (1.95 - 2.95) | (2.22 - 3.05) | (1.88 - 2.9) | (2.24 - 3.07) | (1.9 - 2.88) | (2.24 - 3.06) | (1.9 - 2.88) | (2.24 - 3.08) | (1.88 - 2.92) | (1.83 - 3.75) | (1.46 - 3.58) |  |
| **India** | 3.80 | 3.48 | 4.06 | 3.59 | 3.86 | 3.43 | 3.76 | 3.45 | 3.65 | 3.31 | 3.69 | 3.29 | 3.71 | 3.24 | 3.71 | 3.17 | 3.71 | 3.03 |  |
|  | (4.01 - 3.61) | (3.55 - 3.36) | (4.29 - 3.85) | (3.69 - 3.49) | (4.06 - 3.68) | (3.56 - 3.31) | (3.97 - 3.57) | (3.58 - 3.3) | (3.88 - 3.44) | (3.45 - 3.13) | (3.92 - 3.48) | (3.46 - 3.11) | (3.92 - 3.52) | (3.39 - 3.13) | (3.87 - 3.57) | (3.28 - 3.05) | (2.73 - 5.02) | (2.15 - 4.03) |  |
| **Nepal** | 3.77 | 3.70 | 3.84 | 3.78 | 3.84 | 3.65 | 3.86 | 3.63 | 3.80 | 3.43 | 3.83 | 3.45 | 3.83 | 3.44 | 3.85 | 3.45 | 3.85 | 3.40 |  |
|  | (3.29 - 4.32) | (3.06 - 4.33) | (3.3 - 4.42) | (3.02 - 4.52) | (3.32 - 4.42) | (2.93 - 4.44) | (3.32 - 4.47) | (2.85 - 4.45) | (3.28 - 4.31) | (2.74 - 4.22) | (3.29 - 4.33) | (2.76 - 4.28) | (3.32 - 4.32) | (2.79 - 4.27) | (3.27 - 4.38) | (2.77 - 4.34) | (2.71 - 5.27) | (2.23 - 5.18) |  |
| **Pakistan** | 3.14 | 4.54 | 3.18 | 4.65 | 3.16 | 4.60 | 3.18 | 4.65 | 3.13 | 4.52 | 3.10 | 4.49 | 3.10 | 4.47 | 3.09 | 4.46 | 3.08 | 4.40 |  |
|  | (2.84 - 3.43) | (4.15 - 4.84) | (2.83 - 3.53) | (4.31 - 5.01) | (2.78 - 3.53) | (4.16 - 5.02) | (2.79 - 3.55) | (4.2 - 5.12) | (2.75 - 3.61) | (4.13 - 5.12) | (2.75 - 3.46) | (4.03 - 4.87) | (2.78 - 3.5) | (4.08 - 4.92) | (2.69 - 3.57) | (4.01 - 5.08) | (1.98 - 4.68) | (2.82 - 6.83) |  |
|  |  |  |  |  |  |  |  |  |  |  |  |  |  |  |  |  |  |  |  |
| **Overall** | 3.17 | 3.44 | 3.27 | 3.53 | 3.21 | 3.45 | 3.20 | 3.45 | 3.18 | 3.34 | 3.19 | 3.33 | 3.20 | 3.30 | 3.20 | 3.29 | 3.20 | 3.21 |  |
|  | (2.92 - 3.41) | (3.07 - 3.76) | (3 - 3.54) | (3.17 - 3.88) | (2.93 - 3.49) | (3.06 - 3.81) | (2.91 - 3.49) | (3.04 - 3.84) | (2.88 - 3.48) | (2.96 - 3.76) | (2.89 - 3.46) | (2.94 - 3.7) | (2.9 - 3.48) | (2.93 - 3.71) | (2.86 - 3.52) | (2.88 - 3.75) | (2.22 - 4.49) | (2.14 - 4.75) |  |
| **South Asia average** | 3.17 | 3.38 | 3.28 | 3.48 | 3.22 | 3.40 | 3.21 | 3.40 | 3.19 | 3.29 | 3.20 | 3.28 | 3.21 | 3.26 | 3.21 | 3.24 | 3.55 | 3.11 |  |
|  | (2.93 - 3.42) | (3.03 - 3.71) | (3.02 - 3.55) | (3.12 - 3.84) | (2.95 - 3.5) | (3.02 - 3.77) | (2.93 - 3.5) | (2.99 - 3.79) | (2.9 - 3.49) | (2.91 - 3.71) | (2.91 - 3.48) | (2.9 - 3.67) | (2.91 - 3.49) | (2.89 - 3.68) | (2.88 - 3.53) | (2.84 - 3.71) | (2.68 - 4.67) | (2.27 - 4.03) |  |

| **Supplemental Table 40: Women to men ratio (95% UI)** | | | | | | | | | | | | | | | | | | |  |  |
| --- | --- | --- | --- | --- | --- | --- | --- | --- | --- | --- | --- | --- | --- | --- | --- | --- | --- | --- | --- | --- |
|  |  |  |  |  |  |  |  |  |  |  |  |  |  |  |  |  |  |  |  | |
| **Country** | **2005** | **2006** | **2007** | **2008** | **2009** | **2010** | **2011** | **2012** | **2013** | **2014** | **2015** | **2016** | **2017** | **2018** | **2019** | **2020** | **2021** |  | |  |
|  |  |  |  |  |  |  |  |  |  |  |  |  |  |  |  |  |  |  | |  |
| **Bangladesh** | 0.89 | 0.86 | 0.86 | 0.88 | 0.90 | 0.92 | 1.00 | 1.10 | 1.10 | 1.09 | 1.10 | 1.09 | 1.03 | 1.02 | 1.01 | 1.00 | 0.97 |  | |  |
|  | (0.51 - 1.49) | (0.78 - 0.93) | (0.78 - 0.91) | (0.8 - 0.93) | (0.82 - 0.94) | (0.85 - 0.98) | (0.95 - 1.04) | (1.08 - 1.11) | (1.06 - 1.12) | (1.06 - 1.1) | (1.07 - 1.13) | (1.04 - 1.1) | (1 - 1.05) | (0.99 - 1.05) | (0.99 - 1.04) | (0.98 - 1.03) | (0.48 - 2.02) |  | |  |
| **Bhutan** | 1.03 | 1.01 | 0.99 | 0.97 | 0.95 | 0.94 | 0.93 | 0.94 | 0.93 | 0.94 | 0.93 | 0.92 | 0.90 | 0.90 | 0.89 | 0.89 | 0.88 |  | |  |
|  | (0.48 - 2.20) | (0.98 - 1.03) | (0.96 - 1.01) | (0.94 - 1) | (0.92 - 0.98) | (0.89 - 0.98) | (0.9 - 0.99) | (0.89 - 1) | (0.88 - 0.99) | (0.9 - 0.98) | (0.87 - 0.96) | (0.86 - 0.97) | (0.85 - 0.95) | (0.85 - 0.94) | (0.85 - 0.94) | (0.84 - 0.95) | (0.39 - 1.96) |  | |  |
| **India** | 0.91 | 0.94 | 0.93 | 0.92 | 0.91 | 0.88 | 0.88 | 0.92 | 0.91 | 0.88 | 0.89 | 0.92 | 0.91 | 0.89 | 0.87 | 0.85 | 0.82 |  | |  |
|  | (0.54 - 1.48) | (0.9 - 0.95) | (0.89 - 0.94) | (0.89 - 0.93) | (0.88 - 0.93) | (0.84 - 0.89) | (0.85 - 0.9) | (0.9 - 0.95) | (0.89 - 0.93) | (0.86 - 0.91) | (0.88 - 0.9) | (0.9 - 0.93) | (0.89 - 0.91) | (0.88 - 0.89) | (0.87 - 0.89) | (0.85 - 0.85) | (0.43 - 1.48) |  | |  |
| **Nepal** | 1.17 | 1.14 | 1.12 | 1.09 | 1.06 | 1.04 | 1.01 | 1.01 | 0.98 | 0.99 | 0.95 | 0.94 | 0.90 | 0.90 | 0.90 | 0.90 | 0.88 |  | |  |
|  | (0.66 - 2.14) | (1.14 - 1.16) | (1.14 - 1.13) | (1.09 - 1.12) | (1.05 - 1.08) | (1.02 - 1.08) | (0.97 - 1.03) | (0.97 - 1.03) | (0.93 - 1) | (0.91 - 1.02) | (0.88 - 1) | (0.86 - 1) | (0.84 - 0.98) | (0.84 - 0.99) | (0.84 - 0.99) | (0.85 - 0.99) | (0.42 - 1.91) |  | |  |
| **Pakistan** | 1.51 | 1.48 | 1.45 | 1.43 | 1.41 | 1.40 | 1.40 | 1.42 | 1.44 | 1.46 | 1.46 | 1.46 | 1.44 | 1.45 | 1.44 | 1.44 | 1.43 |  | |  |
|  | (0.83 - 2.91) | (1.52 - 1.44) | (1.49 - 1.4) | (1.51 - 1.38) | (1.44 - 1.38) | (1.46 - 1.37) | (1.47 - 1.34) | (1.47 - 1.4) | (1.46 - 1.41) | (1.52 - 1.42) | (1.5 - 1.42) | (1.51 - 1.44) | (1.5 - 1.42) | (1.46 - 1.41) | (1.47 - 1.41) | (1.49 - 1.42) | (0.60 - 3.46) |  | |  |
|  |  |  |  |  |  |  |  |  |  |  |  |  |  |  |  |  |  |  | |  |
| **Overall** | 1.11 | 1.10 | 1.08 | 1.07 | 1.06 | 1.05 | 1.06 | 1.09 | 1.08 | 1.08 | 1.07 | 1.08 | 1.05 | 1.04 | 1.03 | 1.03 | 1.00 |  | |  |
|  | (0.62 - 2.02) | (1.07 - 1.11) | (1.06 - 1.09) | (1.05 - 1.09) | (1.03 - 1.08) | (1.02 - 1.07) | (1.04 - 1.08) | (1.07 - 1.11) | (1.05 - 1.1) | (1.05 - 1.1) | (1.04 - 1.09) | (1.04 - 1.1) | (1.03 - 1.08) | (1.01 - 1.07) | (1.01 - 1.07) | (1.01 - 1.06) | (0.48 - 2.14) |  | |  |
| **South Asia average** | 0.96 | 1.09 | 1.07 | 1.06 | 1.05 | 1.04 | 1.04 | 1.08 | 1.08 | 1.07 | 1.07 | 1.07 | 1.04 | 1.03 | 1.02 | 1.02 | 0.87 |  | |  |
|  | 0.60 - 1.52) | (1.07 - 1.1) | (1.05 - 1.08) | (1.05 - 1.08) | (1.02 - 1.07) | (1.01 - 1.06) | (1.03 - 1.06) | (1.06 - 1.1) | (1.05 - 1.09) | (1.05 - 1.09) | (1.04 - 1.08) | (1.04 - 1.09) | (1.02 - 1.06) | (1.01 - 1.06) | (1 - 1.05) | (1 - 1.05) | (0.49 - 1.51) |  | |  |

# **Detailed methodology and data sources: GATHER Statement^1^**

| **Item number** | **Checklist item** | **Reporting location** |
| --- | --- | --- |
| **Objectives and funding** | | |
| 1 | Define the indicator(s), populations (including age, sex, and geographic entities), and time period(s) for which estimates were made. | Main text methods |
| 2 | List the funding sources for the work. | Main text (methods section); primary funder given in the “role of the funding source” section |
| **Data inputs** | | |
| *For all data inputs from multiple sources that are synthesised as part of the study:* | | |
| 3 | Describe how the data were identified and how the data were accessed. | Main text methods, with references provided for access to full details |
| 4 | Specify the inclusion and exclusion criteria. Identify all ad-hoc exclusions. | References provided in the main text methods for access to full details |
| 5 | Provide information about all included data sources and their main characteristics. For each data source used, report reference information or contact name/institution, population represented, data collection method, year(s) of data collection, sex and age range, diagnostic criteria or measurement method, and sample size, as relevant. | Overview in main text methods; citations for all references available at  <http://ghdx.healthdata.org/gbd-2021/sources> and  <https://fred.stlouisfed.org/series/NYGDPPCAPCDSAS> |
| 6 | Identify and describe any categories of input data that have potentially important biases (eg, based on characteristics listed in item 5). | Study limitations subsection in discussion, with references provided for access to full details |
| *For data inputs that contribute to the analysis but were not synthesised as part of the study:* | | |
| 7 | Describe and give sources for any other data inputs. | N/A |
| *For all data inputs:* | | |
| 8 | Provide all data inputs in a file format from which data can be efficiently extracted (eg, a spreadsheet rather than a PDF), including all relevant meta-data listed in item 5. For any data inputs that cannot be shared because of ethical or legal reasons, such as third-party ownership, provide a contact name or the name of the institution that retains the right to the data. | <http://ghdx.healthdata.org/gbd-2021/sources>  Availability of data is dependent on data use agreements; contact information provided when not directly available. |
| **Data analysis** | | |
| 9 | Provide a conceptual overview of the data analysis method. A diagram may be helpful. | Main text methods, with references provided for access to full details |
| 10 | Provide a detailed description of all steps of the analysis, including mathematical formulae. This description should cover, as relevant, data cleaning, data pre-processing, data adjustments and weighting of data sources, and mathematical or statistical model(s). | Main text methods, statistical analysis, with references provided for access to full details |
| 11 | Describe how candidate models were evaluated and how the final model(s) were selected. | References provided in the main text methods for access to full details |
| 12 | Provide the results of an evaluation of model performance, if done, as well as the results of any relevant sensitivity analysis. | N/A |
| 13 | Describe methods of calculating uncertainty of the estimates. State which sources of uncertainty were, and were not, accounted for in the uncertainty analysis. | Main text methods |
| 14 | State how analytical or statistical source code used to generate estimates can be accessed. | <http://ghdx.healthdata.org/gbd-2021/code> |
| **Results and discussion** | | |
| 15 | Provide published estimates in a file format from which data can be efficiently extracted. | All estimates are available for download at  <http://ghdx.healthdata.org/gbd-2021/code> and  <https://vizhub.healthdata.org/gbd-compare/> |
| 16 | Report a quantitative measure of the uncertainty of the estimates (eg, uncertainty intervals). | UIs given in all results section, including in figures (as appropriate) and tables |
| 17 | Interpret results in light of existing evidence. If updating a previous set of estimates, describe the reasons for changes in estimates. | Main text discussion |
| 18 | Discuss limitations of the estimates. Include a discussion of any modelling assumptions or data limitations that affect interpretation of the estimates. | Main text study limitations section of discussion |

# **References**

1. **Stevens GA, Alkema L, Black RE, et al.** Guidelines for accurate and transparent health estimates reporting: the GATHER statement. Lancet 2016; 388: e19–23.
2. **Vos T, Lim SS, Abbafati C, et al.** Global burden of 369 diseases and injuries in 204 countries and territories, 1990–2019: a systematic analysis for the Global Burden of Disease Study 2019. Lancet 2020; 396: 1204–22.
3. **Ferrari AJ, Santomauro DF, Aali A, et al.** Global incidence, prevalence, years lived with disability (YLDs), disability-adjusted life-years (DALYs), and healthy life expectancy (HALE) for 371 diseases and injuries in 204 countries and territories and 811 subnational locations, 1990–2021: a systematic analysis for the Global Burden of Disease Study 2021. Lancet (in press).
4. **Kim MS, Hwang J, Yon DK, et al.** Global burden of peripheral artery disease and its risk factors, 1990–2019: a systematic analysis for the Global Burden of Disease Study 2019. Lancet Glob Health 2023; 11: e1553–65.
5. **Brauer M, et al.** Global burden and strength of evidence for 88 risk factors in 204 countries and 811 subnational locations, 1990–2021: a systematic analysis for the Global Burden of Disease Study 2021. *Lancet.* 2024;403(10440):2162-2203.
6. **Thygesen K, Alpert JS, Jaffe AS, et al.** Fourth universal definition of myocardial infarction (2018). *J Am Coll Cardiol*2018; 72: 2231–64. doi: 10.1016/j.jacc.2018.08.1038.
7. **Institute for Health Metrics and Evaluation (IHME).** Global Burden of Disease Study 2021: definitions, data, and methods. Available from: [https://www.healthdata.org](https://www.healthdata.org/). Accessed November 18, 2024.
